# Nucleosome retention by histone chaperones and remodelers occludes pervasive DNA–protein binding

**DOI:** 10.1093/nar/gkad615

**Published:** 2023-07-26

**Authors:** Felix Jonas, Matan Vidavski, Eli Benuck, Naama Barkai, Gilad Yaakov

**Affiliations:** Department of Molecular Genetics, Weizmann Institute of Science, Rehovot, Israel; Department of Molecular Genetics, Weizmann Institute of Science, Rehovot, Israel; Department of Molecular Genetics, Weizmann Institute of Science, Rehovot, Israel; Department of Molecular Genetics, Weizmann Institute of Science, Rehovot, Israel; Department of Molecular Genetics, Weizmann Institute of Science, Rehovot, Israel

## Abstract

DNA packaging within chromatin depends on histone chaperones and remodelers that form and position nucleosomes. Cells express multiple such chromatin regulators with overlapping *in-vitro* activities. Defining specific *in-vivo* activities requires monitoring histone dynamics during regulator depletion, which has been technically challenging. We have recently generated histone-exchange sensors in *Saccharomyces cerevisiae*, which we now use to define the contributions of 15 regulators to histone dynamics genome-wide. While replication-independent exchange in unperturbed cells maps to promoters, regulator depletions primarily affected gene bodies. Depletion of Spt6, Spt16 or Chd1 sharply increased nucleosome replacement sequentially at the beginning, middle or end of highly expressed gene bodies. They further triggered re-localization of chaperones to affected gene body regions, which compensated for nucleosome loss during transcription complex passage, but concurred with extensive TF binding in gene bodies. We provide a unified quantitative screen highlighting regulator roles in retaining nucleosome binding during transcription and preserving genomic packaging.

## Graphical Abstract

**Graphical Abstract:**
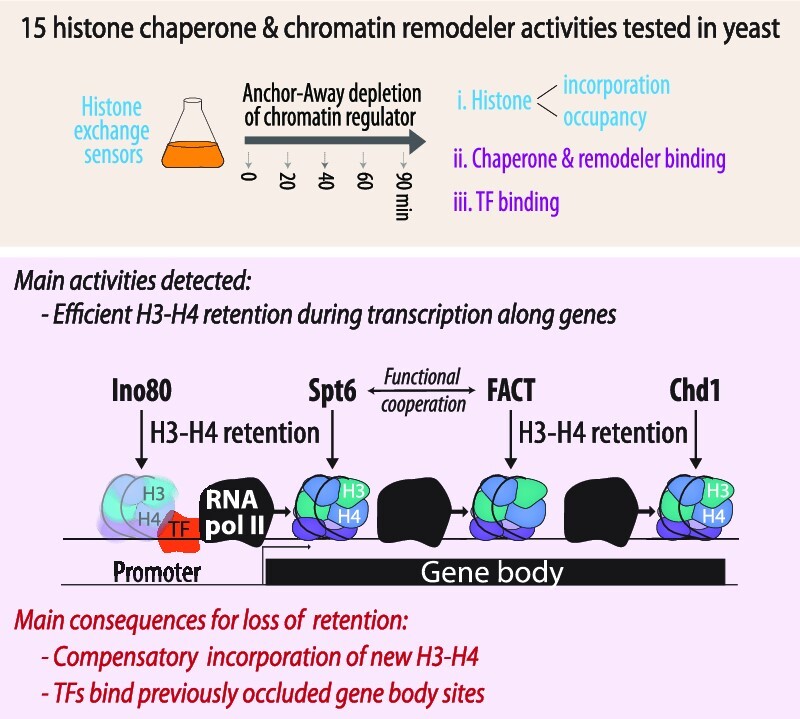

## INTRODUCTION

Histones are small proteins that package eukaryotic genomes within chromatin. At the core of this packaging are nucleosomes, composed of histone octamers wrapped by ∼147 base pairs (bp) of DNA (1). Nucleosomes restrict DNA access and by this protect DNA from damage but also form a barrier for processes that use DNA as a template, including gene transcription and DNA replication (2–4). Enzymes that regulate nucleosome incorporation, stability, positioning or occupancy along DNA shape the nucleosome landscape, exerting genome-wide effects on DNA-related processes.

Under physiological conditions, histones do not form nucleosomes spontaneously and are in fact prone to aggregation. Moreover, being of positive charge, they tend to non-specifically bind DNA (5). To prevent such deleterious interactions, chaperones bind histone proteins throughout their life cycle, from synthesis through nuclear translocation to DNA incorporation (5–9). In particular, histone chaperones guide the ordered assembly of nucleosomes (10) which proceeds through the binding of a histone (H3–H4)_2_ tetramer to DNA, followed by the association of two H2A–H2B dimers (11,12). Once deposited, ATP-dependent chromatin remodelers can slide or evict nucleosomes to adjust their locations along the DNA (13). DNA replication involves the sequential formation of new nucleosomes along the entire genome, to package newly synthesized DNA. The majority of replication-independent exchange localizes to particular regulatory regions, i.e. promoters in yeast and enhancers in mammalian cells (14–16). By contrast, In gene-bodies, H3–H4 is stable or recycled, while replacement with new H2A–H2B occurs readily during transcription (14).

Cells contain about twenty histone chaperones and remodeler complexes. Most of these factors, here collectively referred to as chromatin regulators, are conserved from yeast to human (6,7,17–19). High-resolution structures of chaperones (6,20) and remodelers (21) are available for many of these complexes, both in isolation and when bound to histones or DNA. An emerging picture from structural and functional studies is that histones bind chaperones through the same interfaces used for binding other histones or DNA, pointing at binding competition as a central means through which chaperones direct nucleosome dynamics (6). Successful *in-vitro* reconstitutions have further demonstrated how these regulators can guide the formation of nucleosome arrays and facilitate the progression of the large transcription and replication machineries through chromatin (22–27).

While *in-vitro* analyses have provided much mechanistic and structural understanding of regulator activities, translating these insights into the *in-vivo* context has remained a challenge. Regulators of similar mechanistic capacity can function differently within cells, driving the incorporation, eviction or replacement of histones in promoters or gene bodies, or at particular genomic locations or gene classes. Notably, (H3–H4)_2_ tetramer exchange occurs at gene promoters, but is not evident at gene bodies where H2A–H2B exchange is widespread (14). To what extent chaperones accelerate (H3–H4)_2_ exchange at promoters, or alternatively retain nucleosomes at gene bodies, is not known.

To define the *in-vivo* roles of chromatin regulators, histone exchange along the genome should ideally be measured immediately following regulator depletion. This has been challenging using prevailing methods for measuring histone exchange, which are of limited temporal resolution due to their reliance on pulse-chase histone labeling (16,28–30). Indeed, barring few exceptions (15,31–33), existing studies approximate histone exchange indirectly from nucleosome occupancy, organization, modifications or downstream processes such as transcription or replication (34–47). Further, existing data contain sparse information of where chaperones localize along the genome, confounding the ability for global comparisons.

To overcome these limitations, we apply the pulse-chase-free ‘histone-exchange sensor’ we recently described (14,48), and aim to define the contribution of 15 tested histone chaperones and chromatin remodelers to H3 and H2B histone exchange along the budding yeast genome. We combined the exchange sensor with the anchor-away system (49) to conditionally deplete a given regulator, and further used ChEC-seq (50) to map the genomic binding of all tested regulators. The dominant detected outcome of regulator depletion was an increase in H3 replacement within the gene body. Depletion of Spt6, Spt16 (FACT) and Chd1 sequentially affected H3 retention at the start, middle and end of gene bodies, and also triggered translocation of promoter-residing proteins to the affected gene body region. This re-localization on the one hand ensured sufficient nucleosome binding for genome protection, but also included spurious binding of transcription factors. Together, our data provide a unified quantitative view on the regulation of nucleosome dynamics and indicate nucleosome retention as the preeminent function of the chromatin regulators we tested in the face of transcription.

## MATERIALS AND METHODS

### Yeast strains

Genetic modifications for Anchor-Away (AA, (49)) were inserted into strains carrying the H3 sensor (*HHT2* allele, strain YGY670) and H2B (*HTB2*, YGY688) (14) using a single CRISPR-Cas9 transformation. Specifically, the *tor1-1* point mutation, *fpr1* deletion and the fusion of 2xFKB12 to *RPL13A* were simultaneously inserted using repair PCR fragments (harboring the relevant modification) generated from the original AA strain (HHY168, (49)). We note that the *tor1-1* point mutation in HHY168 (and correspondingly in our strains) in fact corresponds to the *tor2-1* S1971I mutation (51) inserted in the TOR1 gene. Chromatin regulators targeted for AA depletion were individually fused using standard yeast transformation (52) to the human FRB domain, generated by PCR from HHY168 with oligos containing the corresponding 45 bp homology. CRISPR-Cas9 was used to simultaneously fuse FRB to multiple chromatin regulators for their co-depletion in strains with more than one AA target.

Strains for ChEC-seq analysis were generated by C-terminally fusing MNase to the indicated target in the desired AA strain. For Reb1, MNase was N-terminally fused. All strains were verified by Sanger sequencing and per experiment in the associated genomic data. See Supplementary Table S1 for a complete list of strains generated in this study.

### Growth conditions

All experiments were carried out on *S. cerevisiae* cultures with at least 6 exponential doublings, reaching an OD 0.4–0.6 in YPD media at 30°C at the start of the experiment. Cells were taken prior (time 0) and at the indicated time following Rapamycin (BarNaor BNR-5000) addition to a final concentration of 1ug/ml of culture. 50 ml samples were taken for ChIP experiments, crosslinked for 5 min at room temperature (RT) in 1% Formaldehyde (37% stock Baker, Cat. # 7040.1000), quenched by adding 0.125 M freshly prepared Glycine (Merck G7126) at RT for 5 min, washed twice in 50 ml ice-cold water, pelleted and snap frozen in liquid nitrogen. 15 ml samples were taken for ChEC experiments.

### Growth spot tests

Exponentially growing cultures were brought to OD of 0.3 and serially diluted 4-fold. The cultures and five serial dilutions were then spotted onto YPD plates supplemented or not with 1 ug/ml of rapamycin and incubated at the indicated temperature for 2.5 days.

### Chromatin immuno-precipitation (ChIP-seq)

ChIP was carried out as in (14). Cell pellets were thawed on ice and washed in 10 ml 1M sorbitol. After complete liquid removal, pellets were resuspended in 600 ul RIPA buffer (10 mM Tris pH 8.0, 140 mM NaCl, 1 mM EDTA, 0.1% SDS, 0.1% sodium deoxycholate, 1% Triton X-100, EDTA-free protease inhibitor cocktail) and transferred to chilled LoBind Eppendorf microcentrifuge tubes containing ∼500 ul of 0.5 mm zirconium oxide beads (Next Advance, ZrOB05). Cells were processed for 3 cycles in a Bullet Blender 24 (Next Advance) at level 8 for 1 min, with 1 minute on ice between cycles. Debris and lysate were transferred by piercing a hole in the bottom of the tube, placing a clean chilled tube under and centrifuging at 600 × g for 5 s at 4C. The upper tube with the Zirconium beads was discarded and the lysate was hard spun at 17000 × g for 10 min at 4C. The supernatant containing cleaved solubilized 8 × myc tags was discarded, and the pellet was thoroughly resuspended in 100 ul NP buffer (10 mM Tris pH 7.4, 1 M sorbitol, 50 mM NaCl, 5 mM MgCl_2_, 1 mM CaCl_2_ and 0.075% NP-40, freshly supplemented with 1 mM β-mercaptoethanol, 500 μM spermidine, and EDTA-free protease inhibitor cocktail), and warmed to 37°C in a heatblock for 5 min. 100 ul NP buffer supplemented with 40 units of Micrococcal Nuclease (Worthington MNase, LS004798) were added and samples were incubated for 20 min at 37°C. The MNase reaction was stopped by adding 200 ul ice-cold stop buffer (220 mM NaCl, 0.2% SDS, 0.2% sodium deoxycholate, 10 mM EDTA, 2%,Triton X-100, EDTA-free protease inhibitor cocktail.), vortexing and placing on ice. Following a 30-min ice incubation, the MNase-treated lysates were centrifuged at 17 000 × g for 10 min at 4°C. MNase digestion resulted in ∼85% mononucleosomes and ∼15% dinucleosomes, with the latter being filtered out in analysis. This degree of digestion was calibrated per MNase stock, and prevents over-digestion by MNase.

The 400 ul supernatant was divided into two separate wells of a 96-well LoBind Eppendorf plate; 110 ul lysate for anti-HA IP (added 10 ul of 12CA5 hybridoma supernatant) or 110 ul lysate for anti-myc (added 10 ul of 9E10 hybridoma supernatant). The subsequent steps and sequencing libraries were prepared as in (14).

### Chromatin endogenous cleavage (ChEC-seq)

The experiments were performed as described previously (53), based on (50). 15 ml from cultures were pelleted at 1500 g and resuspended in 0.5 mL Buffer A (15 mM Tris pH 7.5, 80 mM KCl, 0.1 mM EGTA, 0.2 mM spermine, 0.5 mM spermidine, 1 × Roche cOmplete EDTA-free mini protease inhibitors, 1 mM PMSF), and transferred to 96 deep-well plate. Rapamycin-treated samples were washed in buffers supplemented with 1ug/ml rapamycin throughout (time 0 samples in buffers without rapamycin). Cells were washed twice more in 960 μl Buffer A, pelleted, and resuspended in 150 μl Buffer A containing 0.1% digitonin. Cells were then transferred to an Eppendorf 96-well plate (Axygen PCR-96-AB-C) for 5 min permeabilization. CaCl_2_ was added to a final concentration of 2 mM for exactly 30 seconds, and 120 μl of stop buffer (400 mM NaCl, 20 mM EDTA, 4 mM EGTA and 1% SDS) were mixed with 120 μl of sample. Proteinase K treatment, nucleic acid extraction and library preparation were performed as previously described (50,54).

For absolute binding experiments (Figure 5D), a culture with Aro80-MNase fusion was grown in parallel to the assayed cultures to use as spike-in. Briefly, Aro80 has only 3 very strong unique target sites, making possible its use as an internal spike-in (see below) (55). An Aro80-MNase strain was grown to reach OD 4 at the time of the experiment and resuspended and washed as above (in buffers without rapamycin). Exactly the same volume was added to each well in the 96 well plate during the 5 minute permeabilization, and CaCl_2_ was added to the mixed sample containing the assayed culture mixed with the Aro80 spike-in as above. The internal amount of Aro80-specific signal enables an absolute quantification of the cultures sampled in parallel.

### ChIP and ChEC-seq read processing

For ChIP-seq experiments, demultiplexed, paired-end reads (read1 51 bp, read2: 51 bp with NovaSeq) were aligned to the *Saccharomyces cerevisiae* genome (R64-1-1) using Bowtie 2 (56) with the following parameters: ‘-p8 ‐‐very-sensitive.’ Aligned read pairs with corresponding fragment sizes between 100 and 200 bases were then selected and used to calculate the genome coverage using BEDTools (57) with the ‘-pc -fs 1 -d’ option, resulting in a single ‘coverage unit’ in the middle of the corresponding fragment. The 100–200 bp limit for the ChIP-seq data serves to filter out di-nucleosomes and only analyze fragments that came from mono-nucleosomes. Mono-nucleosomes predominantly consisted of over 85% reads. Coverage files were imported into MATLAB and normalized to a total coverage of ∼10 Mio across the entire nuclear genome without the ribosomal locus. The normalized coverage of repeats was averaged, and this mean normalized coverage used for most analyses in this paper.

For ChEC-seq experiments, demultiplexed reads were first filtered to remove adapter dimers as well as other artifacts (poly-TATA reads) from the library preparation. Filtered reads were then trimmed to a length of 40bp and aligned to the *Saccharomyces cerevisiae* genome (R64-1-1) using Bowtie 2 (56) using the following parameters: -p8 –end-to-end –very-sensitive –trim-to 40.’ All properly aligned read pairs were then used to calculate the genome coverage using BedTools (57) with the ‘-pc -fs 1 -d’ option, resulting in a single ‘coverage unit’ in the middle of the corresponding fragment.

### ChIP-seq raw data visualization (Figure 1B, Supplementary Figure S2C)

Selected region was divided into 20-bp bins and the mean normalized coverage across the biological repeats in each bin calculated, smoothened along 7 bins and shown.

### Relative change after 90 min of regulator depletion for genomic feature types (Figure 1C, Supplementary Figure S2D)

Yeast genome annotation (58), TSS position (59–62), and nucleosome position data (63), were used to assign genome regions to the indicated features, i.e. promoter: −2 and −1 nucleosome, NDR: region between +1 and −1, 5′ the first 350 bp after the TSS, 3′: the last 350 bp before the TTS, mid: the rest. Then we calculated the mean coverage of each ChIP-seq profile over each feature (∼20 000 features in total: *n* = 3, 4, 6, 251, 17, 188, 256, 23, 2444, 3947, 4776 and 4349 for Histone promoters, rRNA37, rRNA5, ARS, Centromere, tRNA, TY + LTR, telomere, promoter, 5′ mid and 3′ respectively). To determine the relative change during regulator depletion we first selected all features of one type, e.g. 5′ gene, log_2_-transformed their occupancy in all repeats of a certain depletion time course, e.g. spt6 depletion and normalized each to its mean coverage at the starting point (before depletion), i.e. relative occupancy. We then fitted a single line across all the relative occupancies of all selected features over all repeats against the time (in min) after depletion for each repeat. The slope of this line was then multiplied by 90 to estimate the mean relative change of this feature type after 90 min of regulator depletion (indicated as color), if not noted otherwise the Pearson correlation coefficient of this relation (rel. occupancy vs. time) is indicated by the size of the dots.

### Relative change in histone dynamics for different gene groups (Figures 2A, 4A, Supplementary Figure S3B)

As above, but the features (5′, mid and 3′) are further subdivided into 5 groups according to the expression level, taken from (64), (or OPN-score) of the corresponding gene. The expression groups consist of 428 genes (lowest expression), 1766, 3070, 832 and 300 genes with the highest expression.

### OPN score calculation (supplementary figure S3)

OPN scores were calculated as described previously (65). In short, for each gene with known TSS the mean occupancy in the TSS-proximal region (150–0 bp upstream) was divided by the mean occupancy in a more distant region (200–400 bp upstream).

### Relative change in histone dynamics along the metagene (Figures 1D, 2C, 4B, Supplementary Figures S2E, S5C)

We first calculated the metagene coverage for every ChIP-profile. The scaled meta gene is 1350 bp long: 250 bp before the TSS, 350 bp after the TSS, 300 bp in the middle, 350 bp before the TTS and 100 bp after the TTS. The meta gene coverage for all, except the middle part, corresponds to the mean coverage across all genes at the respective position with a known TSS and TTS and minimum length of 700 bp. To calculate the coverage in the middle region, we rescaled the middle region of all genes more than 1050 bp. Therefore, the coverage between 350 bp after the TSS and 350 bp before the TTS was smoothened (10-bp window) and the smoothed coverage at 300 regular intervals selected, i.e. 300 bp for each gene, to calculate the mean coverage in the middle region. This approach better preserves the nucleosome patterning in the start and the end of genes.

These mean meta profiles of biological repeats were then log2-transformed (after adding a pseudo-count of 1) and normalized by the corresponding mean zero timepoint to calculate the relative change shown.

For 2C, 4C and S4C, only highly expressed genes (Top200) and the 90-minute depletion timepoint are selected for the metagene.

### Regulator enrichment on genomic feature types or gene region (Figures 1C, 2A, Supplementary Figure S3B)

After annotating the features on the genome (see above), the mean occupancy of each regulator across all features of one type was calculated, i.e. 12 mean occupancies (one per type) per regulator. Then enrichment of a certain regulator at a feature type is then defined as its relative occupancy when compared to all other chaperones (in log2):

EnrR,T = (log_2_(OccR,T)-mean(log2(OccS,T))/ std(log_2_(OccS,T), where EnrR,T is the enrichment of regulator R at the feature type T, OccR,T is the mean occupancy of regulator R across all features of type T and OccS,T is the mean occupancy at features of type T across all regulators (S).

### Myc or HA level in gene region vs. expression level (Figure 2B, Supplementary Figure S4B)

After determining the mean myc or HA level in the three different gene regions (5′,mid and 3′) for each gene at a certain timepoint/regulator, they are sorted by and plotted against the expression level (64) of the corresponding gene and then smoothed using a sliding window of 1 expression level units. The lines then correspond to all timepoints of a certain regulator-depletion time course.

### Chaperone binding profile along the metagene (Figures 2C, 3A, Supplementary Figures S3C, S5A, B)

Steady-state ChEC-seq profiles along the Top200 expressed genes were normalized for length (see above), aligned by their TSS and the mean across those genes is shown. In Figure 2C, for better comparison each mean profile is scaled so that its minimum and maximum are at zero and one, respectively. In Supplementary Figure S3C, the metagene region stretches from −500 to + 100 around each TSS and the genes are selected based on their OPN scores. DPN: bottom 849 genes, OPN: top 93 genes.

### Relative change in regulator binding (Figures 3B, 5F)

Similar to above, but for ChEC-seq profiles instead of ChIP-seq profiles. Terminator corresponds to the 100bp downstream of the TTS. Moreover, instead of calculating the linear fit across all regions kept separately, we first calculated the mean occupancy across all regions of the same type, e.g. 5′ and high expression level. As before, these mean occupancies were then log2-transformed and normalized against mean before fitting a line were the x-value is the time of depletion (0 or 60 min) and the y-value is the mean normalized occupancy. Each biological repeat is one point. The slope x 60 min is then used as the mean relative occupancy change across all repeats.

### Principal component analysis of genome-wide myc changes (Figure 4C)

Each gene was divided into three segments (3′, mid, 5′), i.e. ∼15 000 segments in total, and the mean myc level of each segment in the corresponding samples (myc epitope of the Spt6, Spt16 and Spt6 + Spt16 time courses) calculated and log_2_-transformed. The relative change of each segment in each sample was then calculated by comparing its myc level against the average myc level across all samples. For each sample, relative changes across all segments were z-score normalized and then used as an input for the principal component analysis. The mean values and standard error of the first and second PC across all biological repeats were used to plot Figure 4C.

### TF promoter preference similarity during regulator depletion (Figure 5B)

Promoters of all genes were determined as described previously (66), i.e. from the TSS to the closest upstream verified open reading frame but at least 700 bp. The total sum of normalized reads on each promoter is considered as the promoter binding signal of a TF sample (different TFS and different timepoints) on the respective promoter. The correlation (Pearson's R) between all promoter binding signals (*n* = 5424) of two TF samples are considered as the promoter preference similarity and shown in Figure 5A.

### TF binding signal around motifs during regulator depletion (Figure 5C, Supplementary Figure S6A)

For each TF (Msn2, and Reb1) motif PWM was downloaded from CISBP (67) (ID: M00036_2.00 and M00063_2.00) and all motif occurrences determined with FIMO (68). Based on their genomic location, motifs were assigned to telomeres, gene bodies, ribosomal rRNA, or promoters (= intergenic regions). Mean normalized ChEC across all biological repeats was calculated, the motifs ordered by their occupancy and their type (promoter or gene body) and the occupancy around (+/− 80bp) each motif is shown. For the mean profile, the mean normalized coverage around all motifs of one type, i.e. gene body or promoter, was calculated and is shown.

### Absolute TF binding in promoters and gene bodies during regulator depletion (Figure 5D)

Based on its binding profile, the Aro80 transcription factor has only three target sites (55) and no overlaps with either Reb1 or Msn2 (Pearson's *R* ≤ 0.06 and < 0.03). To normalize each spiked-in TF sample against the Aro80 spike-in, we thus first calculated the normalized number of reads falling into the unique Aro80 binding regions, i.e. spike in reads. Next, we calculated the number of unique TF reads as the mean occupancy around all promoter or gene body motifs (from −25 to −6 and +6 to +25 bp excluding the exact binding site). For each spiked-in sample, these mean occupancies (promoter and gene body) were then divided by the sum of spike in reads and shown as a function of regulator depletion time in Figure 5D.

### Relative TF binding in gene body and promoters (Figure 5E and Supplementary Figure S6B)

To determine the relative binding to gene body versus promoter motifs, the mean normalized occupancy across all promoter or gene body motifs was calculate and divided by each other. The log_2_-transformed ratio is shown.

For Supplementary Figure S6B, the mean occupancy across all promoter or gene body positions (not only motifs) is used.

### Relative TF binding change in certain gene regions and expression levels (Figure 5F)

Motif occurrences were grouped according to their genic position, i.e. promoter, 5′, mid, 3′ or terminator, and the corresponding gene expression level (5 groups as above). For each group, the mean change across all time courses and time points was calculated using a linear fit as described above for myc and HA levels.

### Sensor epitope level around bound and unbound motifs (Figure 5G, Supplementary Figure S6C)

Bound promoter and gene body motifs after regulator depletion were determined by comparing the mean TF occupancy around them after 90 min of regulator depletion against the mean occupancy at a promoter or gene body position. Motifs with a two-fold higher occupancy are considered as bound motifs, and were then grouped into four groups: promoter-bound, gene body-bound, promoter-unbound and gene body-unbound. For each time point the mean HA (or myc) level of the H3 sensor around (+/− 75 bases) all motifs of each of these 4 groups was calculated. Shown is the level for each time point the mean HA (or myc) level on bound (or unbound) gene body motifs against the HA (or myc) of the corresponding promoter motifs.

### Genome-wide similarity between ChEC or ChIP samples (Supplementary Figure S2A, B)

Mean ChEC and ChIP profiles were smoothed using a running average of 141 or 250 bp, respectively. The similarity was calculated as the Pearson's correlation across all promoter and gene body positions between two smoothened profiles.

### H3K79me3 enrichment across metagenes (Supplementary Figure S4D)

Normalized reads along unscaled gene body (500 bp upstream to 1000 bp downstream) of the respective groups (mid and high expressing) are aligned by their TSS, and their mean HA and myc level at the corresponding sample calculated. After smoothing this meta gene profile with a moving average running window of 51 bp, the enrichment at each position is calculated as the log2-transformed ratio between the smoothened, mean H3K79me3 level at this position and the corresponding HA level (after adding a pseudo count of 0.5).

### Expression analysis

Exponentially growing cells were supplemented with 1 ug/ml of rapamycin and harvested every 10 min at the indicated times for total mRNA extraction. RNA was extracted and libraries were prepared and processed as previously described in (14). For the analysis, genes were binned in the same gene groups described above, and the median log_2_ fold change at each time point compared to the steady state condition calculated. Shown is the moving average over three time points of this median.

## RESULTS

**Figure 1.**
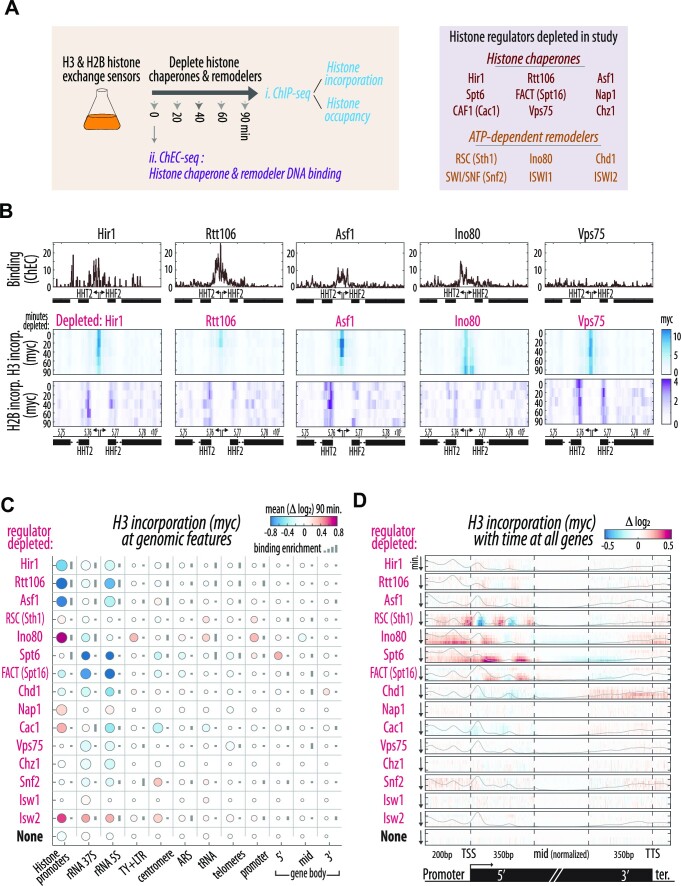
A unified screen to measure yeast histone chaperone and remodeler activities. (**A**) Histone occupancy and incorporation were measured by ChIP-seq as described previously (14) during the course of induced nuclear depletion (Anchor Away, AA) of 15 histone chaperones or remodelers (‘chromatin regulators’, depleted subunit of complex in parentheses). Chromatin regulator localization was measured by ChEC-seq (see Supplementary Figure S2A). (**B**) Histone gene promoter exchange is specifically regulated. Regulator binding (ChEC) and H3 or H2B incorporation levels (myc, ChIP) at the HHT2-HHF2 histones locus during the depletion of the five indicated regulators. See Supplementary Figure S2C for HA. (**C**) Chromatin regulator activities and localizations map to specific genomic features. Mean relative change of myc signal during the 90-minute depletion of each regulator at the indicated genomic features: Histone promoters (includes the regulated HHF/T1, HHF/T2 & HTA/B1 promotors), TY + LTR; TY elements and Long Terminal Repeats, ARS; Autonomous replication sequences, promoters (nucleosomes −2, −1 and NDR) and the 5′ (nucleosomes +1 & +2), middle (+3 on) and 3′ (last 2 nucleosomes) of gene bodies. The size of the circle represents the correlation between relative change and depletion time across all timepoints and features. The length of the bar to the right of each circle represents enrichment levels of the indicated regulator at that region (see Supplementary Figure S2D for H3 HA and H2B myc and HA, see Methods). Note the bottom row with no regulator (‘none’) fused to FRB for depletion, but all other modifications (AA and sensors) and treatments (rapamycin addition) are identical. Regulators are ordered by manual clustering based on effect. The number of repeats is indicated in Supplementary Table S2; in general, 1 time course (5 samples) during the screen and 2–3 repeats (mean shown here) for regulators studied further (Supplementary Figure S4C). 2–3 repeats of ChEC were performed (Supplementary Figure S2A). (**D**) Incorporation of new H3 is a prominent effect associated with a subset of regulators. Color indicates the mean relative change (log_2_) of H3 incorporation (myc) with time (Y axis) for each regulator depleted across all genes aligned to the TSS. The gene body includes 350 bp of its start (generally including nucleosomes +1 & +2) and end (last two nucleosomes), and the middle section which is normalized for length (see Materials and Methods). Steady-state regulator binding along the metagene shown with grey line with Y-axis as binding intensity. No-depletion control is shown on bottom. See Supplementary Figure S2E for H3 (HA) and H2B (myc & HA).

To provide a unified view on the contribution of chromatin regulators to nucleosome dynamics, we screened nine representative chaperones and an additional six remodeler-complexes to detect candidates for deeper exploration (Figure 1A). The selected regulators interact with the core histones H3–H4 (e.g. Asf1, Rtt106, CAF-1 & HIR), H2A–H2B (Nap1, Chz1) or DNA-bound nucleosomes (FACT), and are implicated in transcription (FACT, Chd1, Spt6), replication (CAF-1), histone-variant deposition (Chz1), or combinations thereof. Histone modifiers were not included in this study.

Recently, we introduced a new method—histone exchange timers—for mapping exchange along the genome (14). This method uses a genetically encoded marker that is continuously modified at each genomic locus depending on the local exchange rate. This is achieved by fusing the histone of interest (e.g. H3) to one tag containing two epitopes, myc and HA, connected via a TEV-cleavable linker. Fusing the TEV protease to a second histone (e.g. H2B) enables myc cleavage specifically in the context of DNA-bound nucleosomes where the histone subunits come into proximity. The fraction of uncleaved myc is thus defined by the rate of exchange, with the sensor reporting on histone occupancy (HA) and incorporation rate (myc) along the genome. A single sample of exponentially growing cells is sufficient for defining the genome-wide exchange profile and distinguishing between histone replacement (myc increase), incorporation (HA and myc increase) and eviction (HA reduction). Making use of these sensors previously enabled studying dynamic processes including transcriptional reprogramming and replication (14,48).

To examine how regulator depletion affects histone exchange, we combined the sensors for H3 and H2B histones with the anchor-away system (AA). Briefly, AA depletes the nucleus of a tagged target protein by conditional tethering it to a tagged abundant cytoplasmic anchor protein using rapamycin-dependent heterodimerization (49). Depletion of chromatin regulators led to the expected growth defects (Supplementary Figure S1). We could accordingly confirm the depletion of essential regulators, and non-essential ones that in combination lead to a synthetic growth arrest, but we cannot account for potential differences among regulators in the extent or temporal course of the depletion. We collected samples before and at 20, 40, 60 and 90 min following chaperone depletion, and profiled the two sensor components using Chromatin Immuno-Precipitation (ChIP-seq) (Figure 1A). To help distinguish between direct and indirect effects of regulator depletion, we complemented the exchange data with profiling the localization of all tested regulators across the genome. Since the transient and indirect DNA binding displayed by chromatin regulators is difficult to detect using ChIP-based approaches (69–72), we applied ChEC-seq. ChEC-seq uses a fusion of a protein of interest to micrococcal nuclease (MNase) to target rapid calcium-dependent cleavage to specific genomic loci *in vivo*, providing rapid binding sampling with high sensitivity, reproducibility and spatial resolution (50,53).

### Defining the activity and localization of chromatin regulators across the *S*.*cerevisiae* genome

Overall, we defined the localization and exchange phenotypes of 15 chromatin regulators, representing all major conserved histone chaperones and remodeler complexes except for the H2A.Z-specialized SWR1C (Figure 1A, Supplementary Figure S2A-B). In wild-type cells, the highest H3 exchange rates are found in three of the four bidirectional promoters of histone genes (14). Focusing first on these regions, we confirmed binding of known regulators, Asf1, Hir1 and Rtt106 (73), as compared to others (e.g. Vps75) (Figure 1B, C, Supplementary Figure S2C). Depletion of these factors largely reduced the H3 incorporation rate at the three HIR-regulated histone promoters, consistent with previous observations for Asf1 or HIR deletions (14,74). This effect was specific, as depletion of most other regulators was of little effect at the histone promoters (Figure 1B, C). The only notable exception was Ino80 whose depletion rather increased H3 incorporation at those regulatory regions (Figure 1B, C), possibly having undescribed regulatory roles there. We conclude that the exchange sensor and AA system together are sensitive and efficient enough to capture the specific effects of regulatory chaperones at histone promoters.

To further verify the ability of the sensor to describe differences between the tested regulators, we examined additional genomic features, comparing also to an equivalently modified, grown and treated control with no regulator depleted (Figure 1C, Supplementary Figure S2D). Altered H3 replacement around centromeres was observed upon Snf2 and Cac1 depletion, consistent with known contributions to centromeric nucleosome organization (75–77). Similar specific effects were observed for nucleosomes at the ribosomal RNA (rRNA) loci (altered replacement in Spt6, FACT, Chd1, Isw2 & Ino80, the latter three consistent with (78,79)), and TY elements (Ino80 consistent with (80,81)). Conversely, depletion of the H2A.Z specific chaperone, Chz1, had no discernible effect on exchange at the start of gene bodies where H2A.Z binds (Figure 1C), consistent with the minimal effect of Htz1 deletion (14). Comparing regulator binding and activity signals, we observed overlaps in many cases, but no clear-cut correlation (Figure 1C). We conclude that combining the histone exchange timer with the AA system detects regulator-specific effects on nucleosome exchange patterns.

### Regulators of nucleosome dynamics in gene bodies

**Figure 2.**
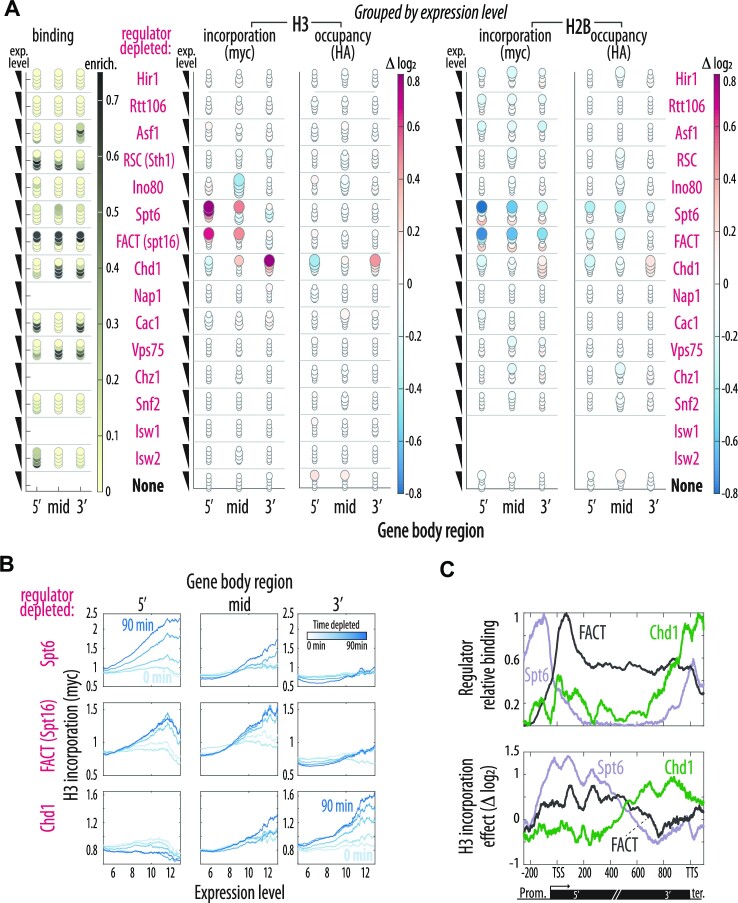
Nucleosomes are actively retained in gene bodies. (**A**) Spt6, FACT and Chd1 repress exchange in highly transcribed gene bodies. Color indicates mean relative change during regulator depletion (log_2_) in H3 & H2B incorporation (myc) & occupancy (HA) for the indicated gene body regions corresponding to Figure 1C, D. Five decreasing expression level bins (highest expression consisting of 300 genes on top) are shown, with circle size corresponding to the *p*-value of correlation between the signal change and depletion time. Mean binding enrichment of each regulator in the corresponding gene body regions on left. See Supplementary Table S2 for number of repeats. (**B**) H3 gene body incorporation increases significantly with time and expression level. Expression dependent (X-axis) mean myc levels for the 5′, middle and 3′ regions of the gene body are shown across all time points (darker are later) following depletion of Spt6, Spt16 or Chd1. See Supplementary Figure S4B for parallel HA analysis, Supplementary Table S2 for number of repeats. (**C**) Spt6, FACT and Chd1 localize and act at the start, middle and end of gene bodies, correspondingly. Top: Rescaled binding is shown to better compare the indicated regulators along the normalized gene body lengths (as above) for the 200 top expressing genes. Bottom: The corresponding relative change in H3 incorporation (myc, log_2_) after 90-min depletion of the respective regulators. See Supplementary Figure S4C for individual repeats.

Focusing on all genes aligned by their Transcription Start Site (TSS) revealed a subset of chaperones whose depletion led to H3 exchange phenotypes at distinct genic regions (Figure 1D, Supplementary Figure S2E). We detected effects of some tested regulators on exchange in gene promoters (most notably Ino80, Figure 1C, D), which we verified through extensive analyses (Supplementary Figure S3). We further observed notable effects of regulator depletions on H3 exchange in gene bodies. In unperturbed cells, H2A–H2B dimers show high replacement, while H3–H4 tetramers are retained (14,16,30,82–84). Analogous to this H2A–H2B exchange, which mainly localizes to highly expressed gene bodies (14), H3 turnover induced during chromatin regulator depletion might most strongly affect a subset of genes. To test this, we classified gene bodies into five groups of increasing expression levels and analyzed the effect of regulator depletion on exchange dynamics in each group (Figure 2A, Supplementary Figure S4A).

Increased H3 replacement in highly expressed genes emerged as the most prominent effect of regulator depletion in gene bodies (Figure 2A). Three chromatin regulators showed this phenotype, including Spt6, a chaperone implicated in transcription elongation, Spt16, a subunit of the Facilitated Chromatin Transcription (FACT) complex, and Chd1, a chromatin remodeler of the CHromodomain-helicase DNA binding (CHD) family (Figure 2A, B, Supplementary Figure S4B). Interestingly, these three regulators acted at different gene body regions with Spt6 at its start, Spt16 more uniformly from the start to the middle, and Chd1 mostly at the 3′ end, similar to their differential localization as determined by ChEC-seq (Figure 2B, C, Supplementary Figure S4B, C).

Of note, many previous studies examining the perturbation of Spt6, FACT (31,35,36,39–41,43,85–88) or Chd1 (44) described transcription-dependent loss and aberration of H3–H4 histones, but we detect substantial effects on nucleosome abundance (HA) only for Chd1 in our experimental setup (Figure 2A, Supplementary Figure S4B). We accordingly performed additional experiments and analyses to evaluate the capacity of our sensor to measure occupancy via HA (Supplementary Note). While we detect an inherent bias of the system that could potentially mask small changes (∼10%), we confirm the capacity of the sensor to detect occupancy changes to an extent more similar to those reported (tens of percent). Taken together, these results suggest that the inconsistency does not result from our inability to measure occupancies. Rather, it may stem from a slower growth rate of our cells, or from regulator inactivation by anchor-away versus a temperature shift used in most other studies.

In our experiments, depletion led most prominently to the expression correlated displacement of ‘old’ H3 histones, which are retained in wild-type cells, with new histones incorporated from the free myc-enriched pool. For Spt6 and FACT, these results are consistent with observations for select genes (31,32), and the global loss of epigenetic marks previously reported (35) (Supplementary Figure S4D). Chd1, FACT and Spt6 have been shown to be bound at gene bodies, but not promoters, of active genes (89,90). Specifically, these three RNA polymerase-associated regulators were reported to be enriched at the start and middle of highly expressed gene bodies. While this is consistent with our measurements for FACT, it differs in the positions where we detect Spt6 and Chd1 binding, perhaps stemming from differences between ChEC and ChIP in capturing proteins associated with the moving polymerase. We note that the relative positioning of Spt6, FACT and Chd1 correspond well to their exchange activities in our measurements (Figure 2C, Supplementary Figure S4C). Further, previous efforts using pulse-chase histone labeling did not detect the prominent effect of Chd1 depletion on the exchange rate (15).

It is notable that the increase in histone replacement caused by chaperone depletion was limited to the H3 histone. In fact, the rapid H2B replacement characterizing highly expressed genes in wild-type cells (14) decreased, rather than increased upon regulator depletion (Figure 2A). Since this decrease extended across the whole gene body and was common to regulators that had different effects on H3 (Figure 2A), it is more difficult to interpret and may stem from a more global effect such as lowering transcription levels. Consistently, when analyzing RNA expression during regulator depletion, we detect a stronger decrease in expression following Spt6 and FACT depletion than in control cells, specifically at highly expressed genes (Supplementary Figure S4E). We conclude that over gene bodies, the critical role of histone chaperones is to prevent transcription-associated loss or replacement of H3–H4 tetramers, with three regulators providing sequential retention along the whole gene-body.

### Binding competition and cooperation within the chromatin regulator network

Our results point at increased H3 replacement along the gene body in highly expressed genes as the prominent and most immediate effect of regulator depletion. To replace H3–H4 histones, nucleosomes must first be evicted, a process that could result from the progressing polymerase, followed by the incorporation of the detected new myc-tagged histones. The fact that no chaperone in our screen led to reduced H3 replacement in gene bodies further supports highly efficient retention in unperturbed cells (14), indicated by the low myc level. The increased incorporation observed upon loss of retention therefore reflects a compensating mechanism not significantly active in unperturbed cells.

**Figure 3.**
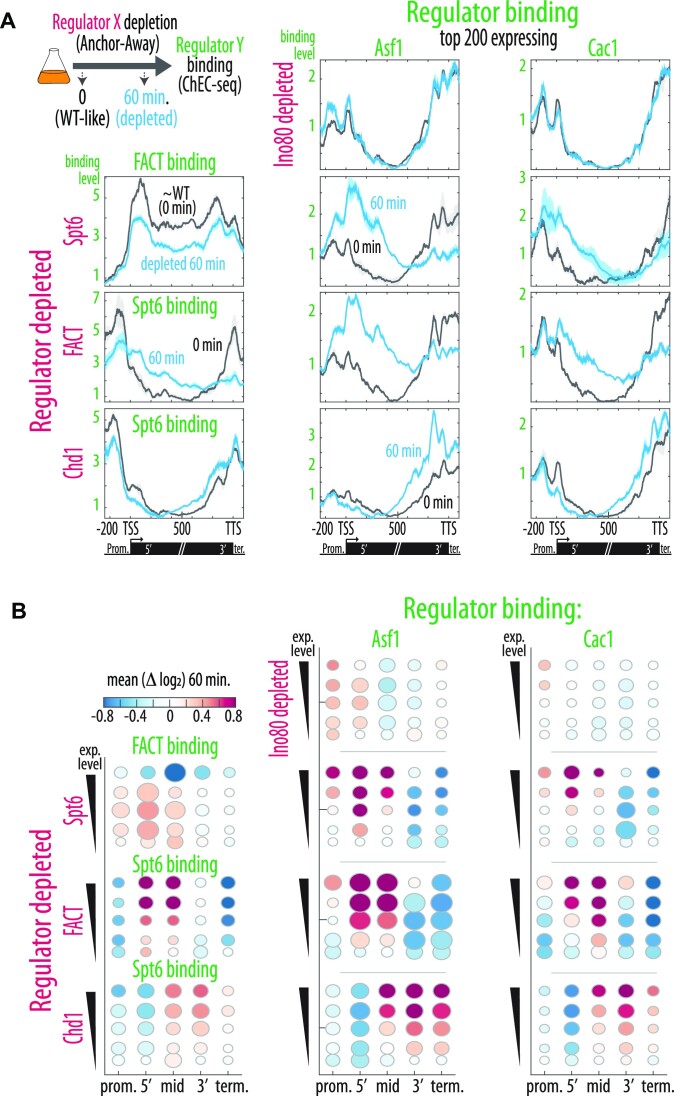
Depletion of key gene body regulators triggers re-localization of promoter-bound regulators to the depleted region. (**A**, **B**) FACT, Spt6, Asf1 and Cac1 re-localize specifically to the region served by the depleted regulator. The indicated chromatin regulator was depleted (magenta; Ino80, Spt6, FACT & Chd1) and binding was measured for other regulators (green; Spt6, FACT, Asf1 & Cac1). Mean binding profiles along the normalized gene body length of the 200 top expressed genes before (black) and following 60 min of depletion (light blue). Note that in the first column, binding is shown for either Spt6 or FACT. Data is the mean of 2–3 repeats (see Supplementary Table S2) with the STDV shaded. (B) Quantification of mean log_2_ change in binding for (A). Regions binned into the 5 gene expression level groups shown for the promoter (250 bp), terminator (100bp) and along the regions of the gene body. Dot size represents the correlation between the signal change and the treatment time across all repeats.

To identify the chaperones that incorporate new histones when H3 retention is lost, we first examined for chaperones that change their localization upon depletion of H3-retaining regulators. We reasoned that to compensate, a chaperone would need to re-localize to the affected locations, previously served by the depleted regulator. Re-localization of Asf1 and Hir1 was previously observed upon depletion of FACT and Spt6, and Asf1 and Hir1 were further suggested to act in re-distributing nucleosomes ejected during transcription (35). We expanded on this in our system, by mapping the binding pattern of five candidate chaperones (Spt6, FACT, Rtt106, Asf1 and Cac1) upon the depletion of each of the four regulators leading to increased H3 replacement: Ino80 in gene promoters (Supplementary Figure S3B-C), and Spt6, Spt16 and Chd1 at different regions of highly expressed genes (Figure 3).

Depletion of the promoter-serving Ino80 (Figure 1D, Supplementary Figure S3B) had no detectable effect on the localization of the tested chaperones, which already primarily reside in the promoter region (Figure 3A). By contrast, depletion of each of the three gene body regulators, Spt6, FACT and Chd1, led to re-localization of all tested chaperones (Figure 3A, B, Supplementary Figure S5A, B). Thus, the promoter localized Asf1, Cac1 and Rtt106 shifted their localization into the gene body of strongly expressed genes, to occupy the specific region previously served by the depleted factor (Figure 3B). Furthermore, localization of Spt6 and FACT themselves changed, but in a manner that suggested binding cooperation rather than competition; Spt6 depletion uniformly reduced occupancy of FACT and more prominently, FACT depletion reduced Spt6 gene body binding while also strongly re-localizing it. We conclude that depletion of the three regulators required for H3 retention in gene bodies leads to a rapid re-localization of other chaperones, which may contribute to the exchange phenotype observed. Further, Spt6 binding depends on FACT binding.

### Functional interactions and redundancy within the chromatin regulator network

**Figure 4.**
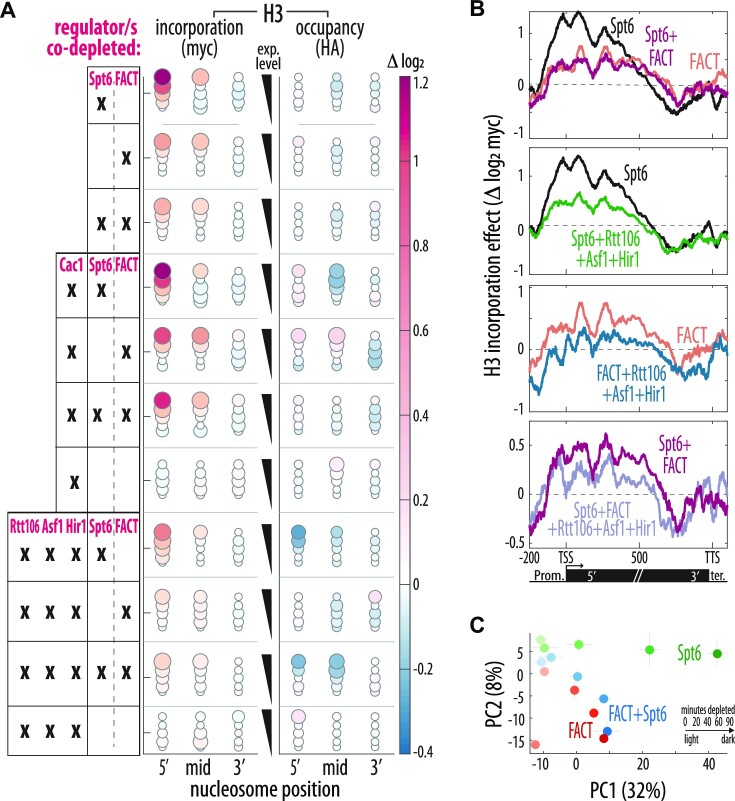
Dependency and competition between chromatin regulator activities. (**A**) Co-depletion of multiple chromatin regulators reveals cooperation and competition. Regulators were depleted individually or in the indicated combinations. Mean relative change after 90 min in the 5 expression level groups for H3 myc and HA signal, i.e. incorporation and occupancy. See Supplementary Table S2 for number of repeats. (**B**) FACT and Spt6 activities are co-dependent, compensatory incorporation relies on Rtt106, Hir1 and Asf1 combined. Relative change of myc along the normalized gene body of highly expressed genes (log_2_) after 90-minute depletion of the indicated regulators and combinations (as Figure 2C). Note that the co-depletion phenotypes indicate that Spt6 activity depends on FACT and vice versa, and that Rtt106, Hir1 and Asf1 co-depletion collectively decreases incorporation in cells depleted of Spt6, FACT or both. See Supplementary Figure S5B for parallel HA analysis. (**C**) FACT & Spt6 co-depletion phenocopies FACT alone. PCA analysis for the relative change in myc levels across all gene bodies regions for all timepoints in Spt6 and FACT depletion individually or together. Color intensity indicates time of depletion. Shown is the mean position and the std error (see Materials and Methods). Note that while the effect of Spt6 depletion is quantitatively stronger, the co-depletion resembles FACT alone.

Our analysis pointed to the effective cooperative and competitive binding among regulators across the genome. To examine the functional role of these interactions, we measured the histone exchange phenotype upon co-depletion of multiple *regulators* (Figure 4A). If a co-depleted *regulator* compensates for the primary chaperone, we expect to lose the compensatory myc integration.

Cells that were co-depleted of Spt6 and FACT still showed increased H3 replacement, but this increase was significantly lower than that observed in Spt6-depleted cells and was near-identical to that observed upon FACT depletion (Figure 4A–C). This is consistent with the reduced Spt6 binding observed upon FACT depletion (Figure 3A-B) and may suggest that FACT cooperates with Spt6 in retaining nucleosomes but accelerates H3 replacement in the absence of Spt6. This dual role is consistent with biochemical assays showing that FACT can promote either chromatin assembly or disassembly depending on the context (91) (See Discussion).

Next, we considered the three promoter-residing chaperones Cac1, Asf1 and Rtt106, which re-localize to gene bodies upon depletion of the gene body-residing regulators Spt6, FACT or Chd1. To examine whether these re-localized chaperones incorporate new histones in these regions, we first depleted Cac1 in parallel to either Spt6, FACT or both. Cac1 co-depletion did not significantly change their effect on histone exchange, suggesting a minor or redundant role of Cac1 in the compensatory histone replacement (Figure 4A). Next, we co-depleted Asf1, Hir1 and Rtt106 in parallel to Spt6, FACT or both. Contrasting Cac1, this co-depletion mostly prevented compensatory replacement (myc) and instead led to reduced histone abundance (HA) (Figure 4A, B, Supplementary Figure S5C). Therefore, Asf1, Hir1 and Rtt106 not only translocate to the locations served by the depleted chaperones, but also compensate reduced histone retention by replenishing lost with new histones.

### Chaperone depletion allows extensive TF binding in coding regions

**Figure 5.**
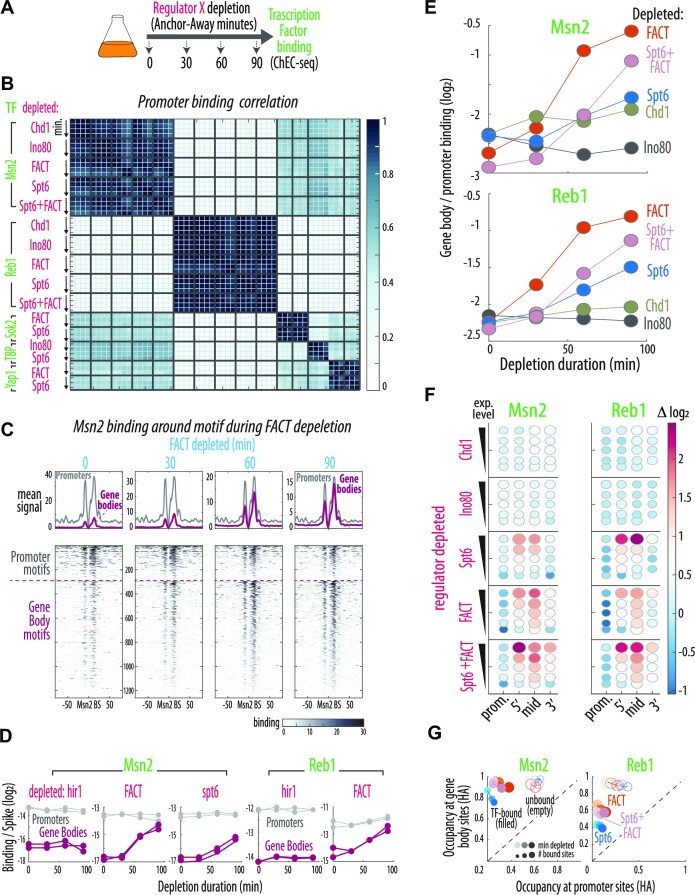
Loss of H3 retention in the gene body provokes high levels of TF binding there. (**A**) An indicated chromatin regulator was depleted (magenta) and DNA binding was measured for indicated factors (green). (**B**) TF promoter preferences remain unchanged during regulator depletion. Indicated regulators that increase H3 exchange were (co-) depleted, and binding of sequence-specific TFs (Msn2, Reb1, Sok2 & Yap1) and TATA box binding protein (TBP) was profiled before and 30, 60 & 90 min following depletion (arrow). Shown is the pairwise correlation of promoter preferences between all samples and all time points. Supplementary Table S2 for number of repeats. (**C**) Msn2 gene body binding reaches levels of promoter binding during FACT depletion. Raw data (bottom) and mean signal (top) centered on the Msn2 *in-vitro* motif (see Methods) at promoters (293 motifs) and gene bodies (magenta for 967 motifs) prior to (‘0′) and 30, 60 or 90 min following Spt16 depletion (see Supplementary Figure S6A for other chaperones and Reb1). (**D**) Regulator depletion provokes increased Msn2 and Reb1 binding at gene bodies. Spike-in normalized motif binding signal of Msn2 and Reb1 during the depletion of the indicated regulators (see Methods). Note that similar binding levels in the promoter versus gene body (see above) are reached by absolute gene body binding increasing with time, rather than promoter binding diminishing. Gene body versus promoter binding remained unchanged in Hir1 depletion, a control regulator with minimal effects on exchange. (**E**) Gene body binding increases to different extents depending on the regulator depleted. Ratio (log_2_) between gene body and promoter mean motif binding for Msn2 (top) and Reb1 (bottom) versus the time of regulator depletion (see Supplementary Figure S6B for Sok2, Yap1 and TBP). (**F**) TF binding change correlates with the gene set (highly expressing) and region served by the depleted regulator. Mean binding change for the 5 expression level groups and indicated gene zones. Dot size indicates *P*-value of correlation. Supplementary Table S2 for number of repeats. (**G**) H3 occupancy in newly bound gene body sites is higher than in bound promoter motifs. Median nucleosome occupancy (HA) around bound and unbound gene body versus promoter TF motifs following regulator depletion. Color shade; min depleted, circle size; number of motifs, filled/empty circles; bound/unbound sites. Note that HA levels remain considerably higher in the newly bound gene body motifs as compared to the promoter, despite the small decrease with time in HA levels at the gene body. See Supplementary Figure S6C for parallel analysis with myc.

To examine whether additional promoter-residing factors translocate to gene bodies upon regulator depletions, we profiled the binding of several DNA binding proteins belonging to different classes: Msn2, Sok2, Yap1 (stress responsive TFs), Reb1 (general TF) and the RNA polymerase II-associated TATA Binding Protein (TBP), following the depletion of Ino80, Chd1, Spt6, Spt16 or their combination (Figure 5A). None of these depletions substantially influenced promoter preferences of any of the tested DNA-binding proteins (Figure 5B).

Broadening our analysis to gene bodies revealed that the depletion of FACT and Spt6, but not Chd1, increased TF binding there (Figure 5C-E, Supplementary Figure S6A-B). Motif-dependent Msn2 binding in the gene body reached equivalent levels to within promoters (Figure 5C). This resulted from increased binding in the gene bodies rather than decreased binding in promoters (Figure 5D). Significant TF binding in gene bodies was observed for Msn2, Reb1 Yap1, Sok2 and TBP when depleting Spt6, FACT or their combination (Figure 5E, Supplementary Figure S6A-B). New gene body binding sites localized preferentially to the *in-vitro* motifs residing in the genes acted on by those regulators, i.e. highly expressed ones (Figure 5F). Of note, this binding within gene bodies occurred in the regulator-dependent regions that showed increased H3 replacement (Figure 5F). Moreover, although binding co-occurred with a minor drop of H3 occupancy, H3 occupancy was still significantly higher in newly bound gene body sites than in bound promoter motifs (Figure 5G). We conclude that lost retention of gene body-bound histones on the one hand allows re-localization of promoter-bound chaperones to those regions to compensate occupancy through the incorporation of new nucleosomes, but on the other hand provokes pervasive TF binding.

## DISCUSSION

The packaging of DNA within chromatin is critical for DNA protection and for orchestrating genome regulation. By depositing, evicting or replacing histones, chaperones and remodelers organize the nucleosome building blocks underlying this packaging. The multiplicity of regulators is subject to extensive studies, both *in-vivo* and *in-vitro*, yet the most immediate effect of regulator action—the nucleosome exchange dynamics—was largely missing from those studies as it was methodologically intractable. Our study now provides a unified measurement of how depleting each of 15 key chromatin regulators impacts histone exchange dynamics at the genomic scale. This was enabled by a recently developed *in-vivo* sensor for histone exchange, which we combined with the anchor-away system for triggering regulator depletion, and high-resolution ChEC-seq for mapping of regulator binding along the genome.

Individual depletion of most regulators led to limited effects on the exchange dynamics when averaged across the genome. These moderate effects could nevertheless be of high functional significance, for example, tuning the precise nucleosome positioning in regulatory regions, as seen for the RSC complex in the context of the +1 nucleosome (Supplementary Figure S3) (72,92–95), or the regulation of particular promoters, as seen for Asf1/Hir1/Rtt106 in the context of the histone promoters (Figure 1B, C) (73). Other regulators, such as Cac1, might act during replication and will therefore show a limited effect in our assay performed on unsynchronized cells, while the function of others might be redundant (47), or might involve specialized conditions such as stress or DNA damage not considered here. We did not detect substantial activity for Vps75, Nap1 or Chz1, but cannot exclude an activity as these depletions were not confirmed in our growth experiment (Supplementary Figure S1).

**Figure 6.**
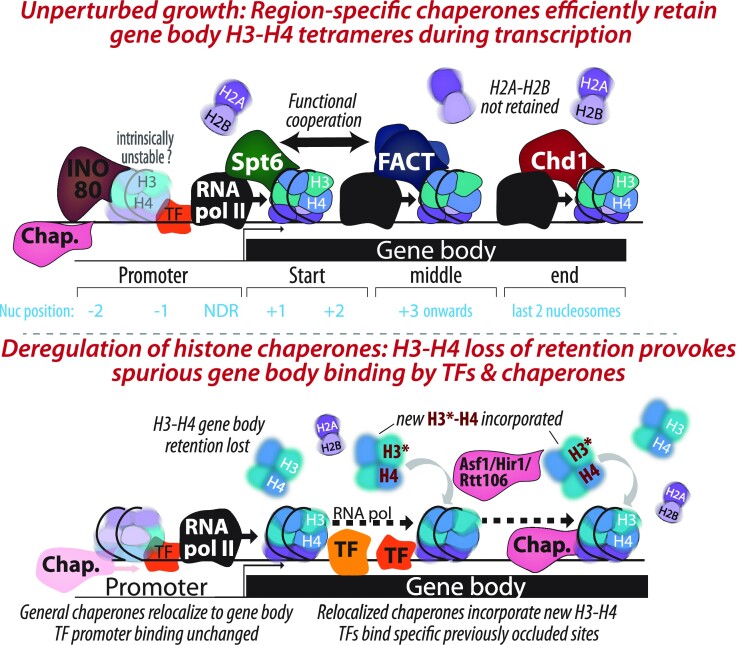
Schematic model In unperturbed conditions (top), key chromatin regulators act at defined gene regions (with the corresponding nucleosome positions annotated below in teal) to retain H3–H4 binding during transcription elongation complex passage. H2A–H2B dimers are not retained, and are replaced with new dimers (14). In the promoter, Ino80 retains H3–H4 in place. For simplicity, regulator interactions with RNA PolII are depicted as direct. Bottom: The depletion of key chromatin regulators (Spt6, FACT or Chd1) results in loss of retention of H3–H4 at their specific region of action, and in the compensatory incorporation of new unbound H3–H4 (red with asterisk) by general regulators (Asf1, Hir1 & Rtt106). Lack of H3–H4 retention in the gene body provokes a re-localization of promoter-binding chaperones (e.g. Asf1, Rtt106 & Hir1), general regulators (e.g. TBP) and Transcription Factors (e.g. Reb1, Msn2) there, without significantly impacting histone occupancy. See ‘Discussion’ for more details.

Notably, four of the tested regulators did affect histone exchange dynamics, and depletion of each of the four resulted in the same effect: increased H3 replacement. This included Ino80, whose depletion increased H3 replacement in gene promoters, and Spt6, Spt16 and Chd1, whose individual depletion increased replacement specifically at the start, middle and end of highly transcribed gene bodies (Figure 6). In all cases, regulator depletion increased incorporation of newly synthesized H3 histones. Our screen has uncovered new potential roles for Spt6 and FACT in increasing exchange at rDNA loci, whereas Isw2 seems to have the opposite activity there (Figure 1C). Similarly, Isw2 and Ino80 may antagonize HIR-induced exchange at histone promoters (Figure 1C), and Ino80 further appears to have a strong H3 retention activity in gene promoters (Supplementary Figure S3). These observations and their biological significance remain to be further characterized.

Our study suggests that *in-vivo*, the role of FACT may depend on the presence of Spt6, so that when Spt6 is present, FACT promotes retention, while in its absence FACT facilitates H3 replacement. This dual role is consistent with biochemical assays showing that FACT can promote either chromatin assembly or disassembly depending on the context (91). In our case, although Spt6 depletion increased H3 replacement to a much higher extent than the depletion of Spt16, co-depletion of both factors was identical to Spt16 alone. An additional indication for functional interactions between FACT and Spt6 include the reduced Spt6 gene body localization we observed upon Spt16 depletion, as well as previous results showing genetic suppression between Spt6 and Spt16 mutations (45). Further studies are required to define the mechanistic basis of this interaction.

Our results suggest that beyond replication, chromatin regulators predominantly act to limit nucleosome replacement. We find this notable for two reasons. First, none of the 15 depletion phenotypes indicated reduced replacement or incorporation. Thus, while four regulators appear to act in nucleosome retention, none of the other regulators act single-handedly in histone incorporation. This suggests that wild-type cells devote much effort to retain the original nucleosomes incorporated during replication throughout the rest of the cell cycle, and that no regulator is solely responsible for the observed ∼300 promoter-localized exchange events per minute (14). Notably, at least within gene bodies, this efficient H3–H4 retention enables the accumulation of regulated and stable epigenetic histone modification patterns, consistent with some marks being lost in Spt6 and Spt16 mutants (35).

The second notable aspect of the observed phenotype is that when retention fails, we do not detect significantly lower histone occupancy. As discussed above, we acknowledge that these results contrast the literature, but do not believe they stem from our inability to measure significant occupancy changes in our system (Supplementary Note). Lower occupancy is expected if incorporation is unlikely when nucleosomes are evicted, but not retained, during gene transcription or TF binding. Our interpretation is that nucleosome eviction was readily compensated through increased incorporation, leading to H3 replacement rather than loss. Depleting Spt6, Spt16 or Chd1 led not only to the loss of H3 retention in gene bodies of highly expressed genes, but also to the re-localization of promoter-residing factors to the affected regions (Figure 6). We observed this when examining for the compensating chromatin regulators that could explain the increased H3 incorporation upon retention loss. All 3 regulators that we tested, Rtt106, Asf1 and Cac1, shifted their localization to bind within the regions affected by the depleted regulator. Co-depletion of multiple regulators indeed verified that some of the re-localized chaperones account for the compensating H3 incorporation (Asf1, Hir1 and Rtt106). Further experiments revealed that TFs, including TBP, also gained access to typically occluded motifs in those regions following Spt6 or Spt16 depletion. Of note, this behavior could lead to the spurious transcription seen during Spt6/FACT depletion (37,38,85,96).

The increased accessibility of gene bodies to the binding of promoter residing factors provides a dual effect. On the one hand, it allows promoter residing chaperones to compensate for the loss of nucleosomes at highly transcribed genes, thereby guarding genome stability. On the other hand, it allows binding of other factors, including sequence-specific TFs (Figure 6). Of note, while Ino80 and Chd1 depletion cause a loss of H3 retention in promoters and gene body ends, they do not lead to increased TF binding, as observed for Spt6 and FACT. Thus, higher exchange in itself may not be sufficient for pervasive TF binding. Chd1 loss of retention is accompanied by an increase in occupancy at the end of gene bodies (Figure 2A), possibly occluding increased TF binding there. In the case of the Ino80, TF binding may be regulated by different means in promoters (e.g. additional features in OPN architecture) as compared to gene bodies where occlusion by nucleosomes may be the main means of negative regulation. The exact means by which chromatin regulators affect pervasive TF binding remain to be studied.

As nucleosome occupancy and the ensuing chromatin ‘openness’ remain largely unaltered in our measurements, three possibilities come to mind that could make these regions more accessible. First, the nucleosomes might be less organized, although we do not see strong evidence for this in our analysis: we detect loss of organization in RSC, Ino80 or Chd1 depletion (Supplementary Figure S3), the latter of which does not increase accessibility in the gene body (Figure 5). Second, rapid nucleosome replacement in itself may be more permissive for binding of other factors, as compared to retained nucleosomes. Our results do not fully support this hypothesis since when comparing Spt6 and Spt16 quantitatively, the level of replacement inflicted does not correlate with the degree of spurious binding: the former is significantly higher in Spt6 depletion, while the latter is slightly lower. Finally, it may be that binding of the chaperones themselves occludes those regions in wild-type cells. This last explanation is consistent with the high abundance of these factors, e.g. FACT and SPT6 are estimated to reach nucleosome levels (97).

## Supplementary Material

gkad615_Supplemental_Files

## Data Availability

Raw sequencing reads and processed coverage profiles can be found on GEO (GSE231810). MATLAB scripts to analyze the data and regenerate the figures will be made available on GitHub (https://github.com/barkailab/Paper_Yaakov2023; permanent DOI: https://doi.org/10.5281/zenodo.8117230).

## Notes

Present address: Felix Jonas, Constructor University, Bremen, Germany.
