# Supplementary material for: Nucleosome retention by histone chaperones and remodelers occludes pervasive DNA–protein binding
Source: Nucleic Acids Res. 2023 Jul 26;51(16):8496–513. doi: 10.1093/nar/gkad615 (PMC10484674; doi:10.1093/nar/gkad615)
Supplement: gkad615_Supplemental_Files [file gkad615_supplemental_files.zip › Supplementary Material.pdf]

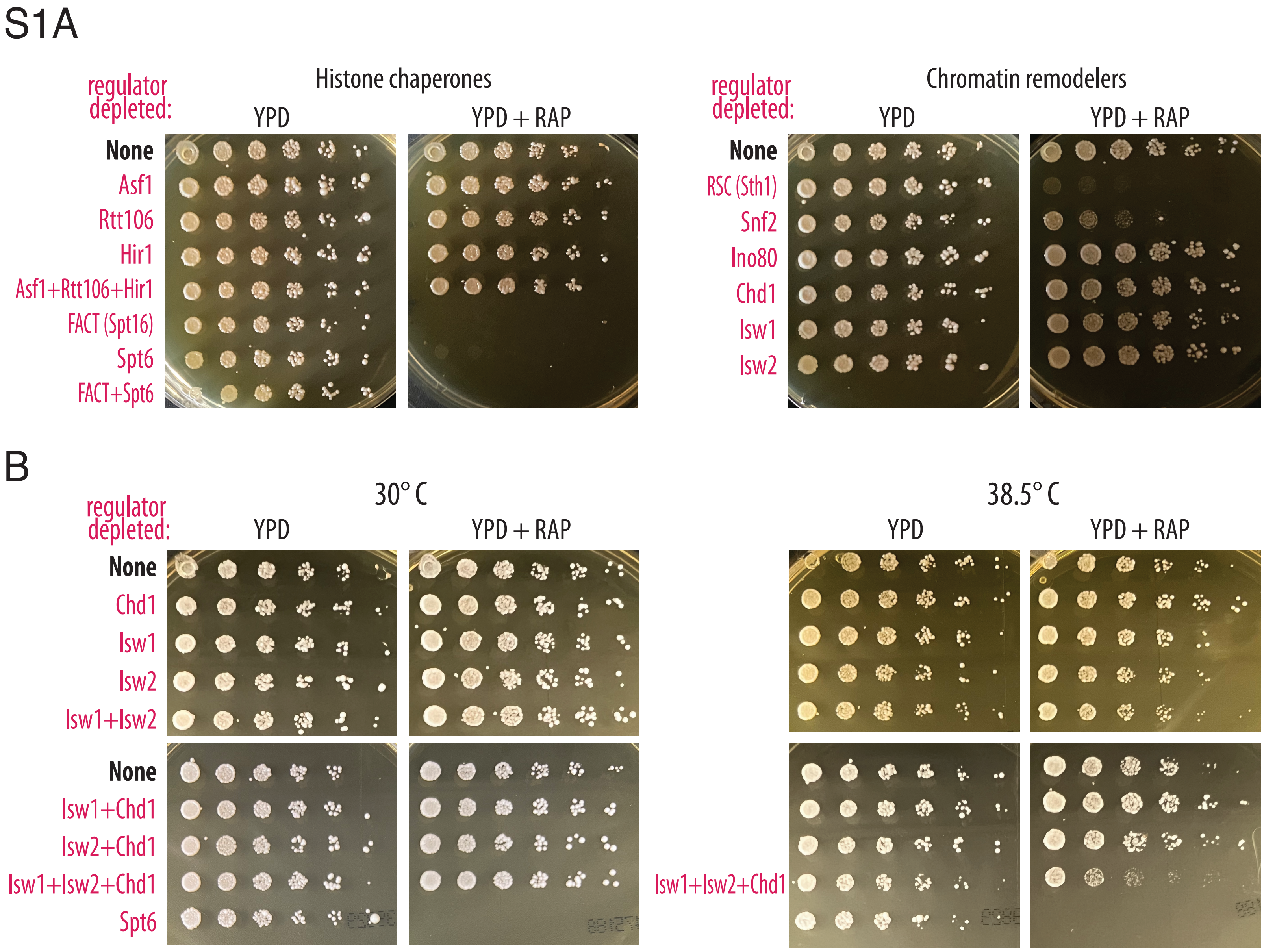
S1A
regulator depleted:
Histone chaperones
YPD
YPD + RAP
None
Asf1
Rtt106
Hir1
Asf1+Rtt106+Hir1
FACT (Spt16)
Spt6
FACT+Spt6
regulator depleted:
Chromatin remodelers
YPD
YPD + RAP
None
RSC (Sth1)
Snf2
Ino80
Chd1
Isw1
Isw2
B
regulator depleted:
30° C
YPD
YPD + RAP
None
Chd1
Isw1
Isw2
Isw1+Isw2
None
Isw1+Chd1
Isw2+Chd1
Isw1+Isw2+Chd1
Spt6
38.5° C
YPD
YPD + RAP
Isw1+Isw2+Chd1

Figure S1. Anchor-away reliably depletes chromatin regulators.

A. Exponentially growing cultures were serially diluted 4-fold, spotted, and grown on YPD plates supplemented or not with 1ug/ml of rapamycin at 30°C as in all liquid-based experiments. Histone chaperones are shown on left, depleted individually or in the indicated combinations. Chromatin remodelers are shown on the right.

B. Co-depletion of regulators that individually have no growth defect leads to synthetic growth defect at elevated temperatures as previously reported (1). Spot tests as above of the indicated chromatin regulator combinations grown at 30°C or 38.5°C. Note the specific growth defect for Isw1+Isw2+Chd1 with rapamycin at 38.5°C. Spt6 is included as a control.

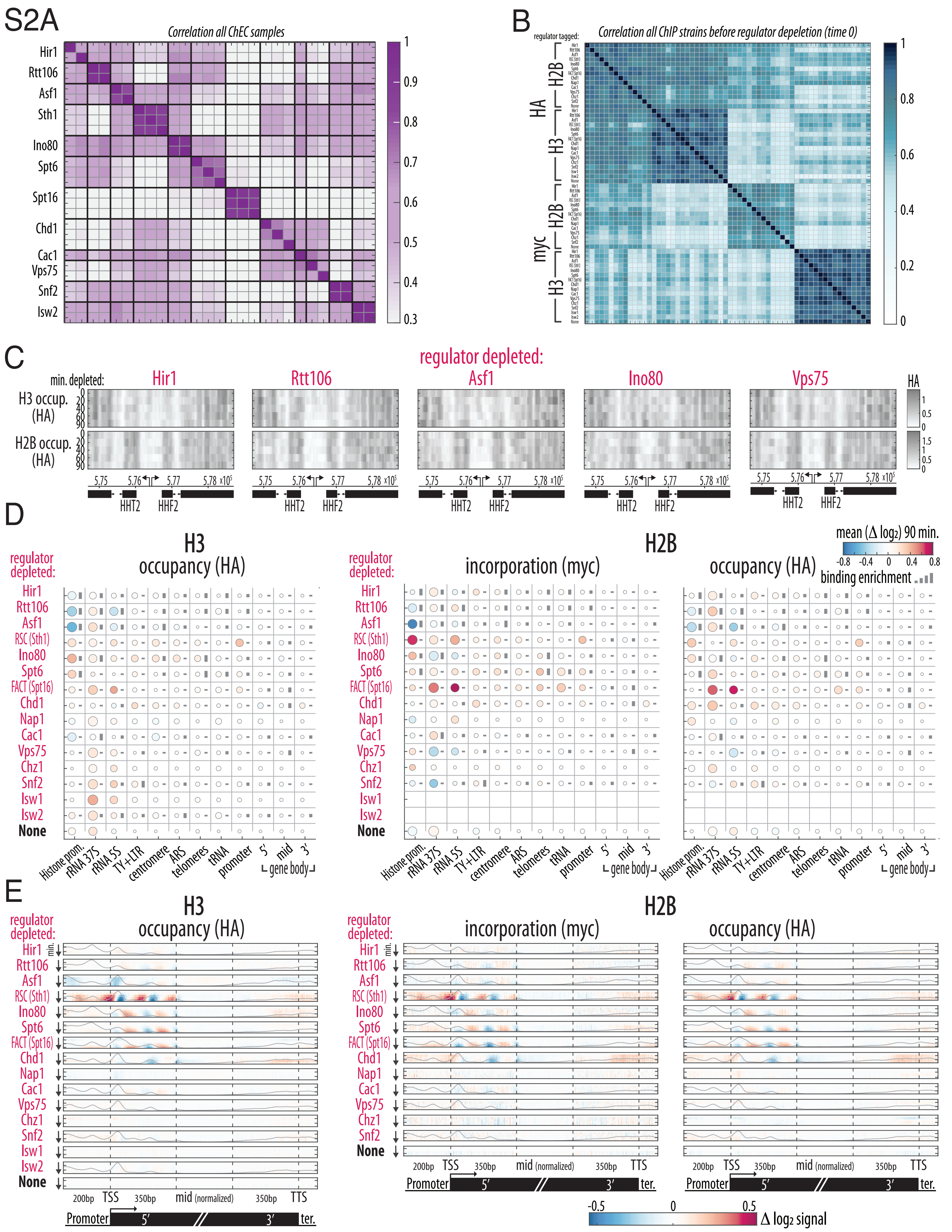
S2A
Correlation all ChEC samples
Hir1
Rtt106
Asf1
Sth1
Ino80
Spt6
Spt16
Chd1
Cac1
Vps75
Snf2
Isw2
B
regulator tagged:
Correlation all ChIP strains before regulator depeletion (time 0)
HA
myc
H2B
H3
C
regulator depleted:
min. depleted:
H3 occup. (HA)
H2B occup. (HA)
Hir1
Rtt106
Asf1
Ino80
Vps75
HHT2
HHF2
D
H3
H2B
occupancy (HA)
incorporation (myc)
regulator depleted:
mean (Δ log2) 90 min.
binding enrichment
Hir1
Rtt106
Asf1
RSC (Sth1)
Ino80
Spt6
FACT (Spt16)
Chd1
Nap1
Cac1
Vps75
Chz1
Snf2
Isw1
Isw2
None
Histone prom.
rRNA 37S
rRNA 5S
TY+LTR
centromere
ARS
telomeres
tRNA
promoter
5'
mid
3'
gene body
E
200bp
TSS
350bp
mid (normalized)
TTS
Promoter
ter.
Δ log2 signal

Figure S2 – A unified screen of yeast histone chaperone and remodeler activities

A. *Correlation across binding measurements for all histone regulators tested*. The indicated histone chaperones and remodelers were assayed by ChEC-seq. The Heatmap showing the pair-wise correlation between individual repeats of all regulators is shown.

B. *Correlation across all histone exchange measurements before histone regulator depletion*. Heatmap comparing the ChIP-seq profiles (Pearson's) of the HA (occupancy) and myc (incorporation) epitopes for H2B and H3 histone exchange timers in all strains at time 0, before the depletion of the indicated histone regulator.

C. Associated with Fig. 1B: HA levels over time for the indicated regulators.

D. Associated with Fig. 1C: Changes across genomic features for histone H3 (HA) and histone H2B (HA and myc).

E. Associated with Fig. 1D: Changes across the metagene profile aligned by the TSS for histone H3 (HA) and histone H2B (HA and myc).

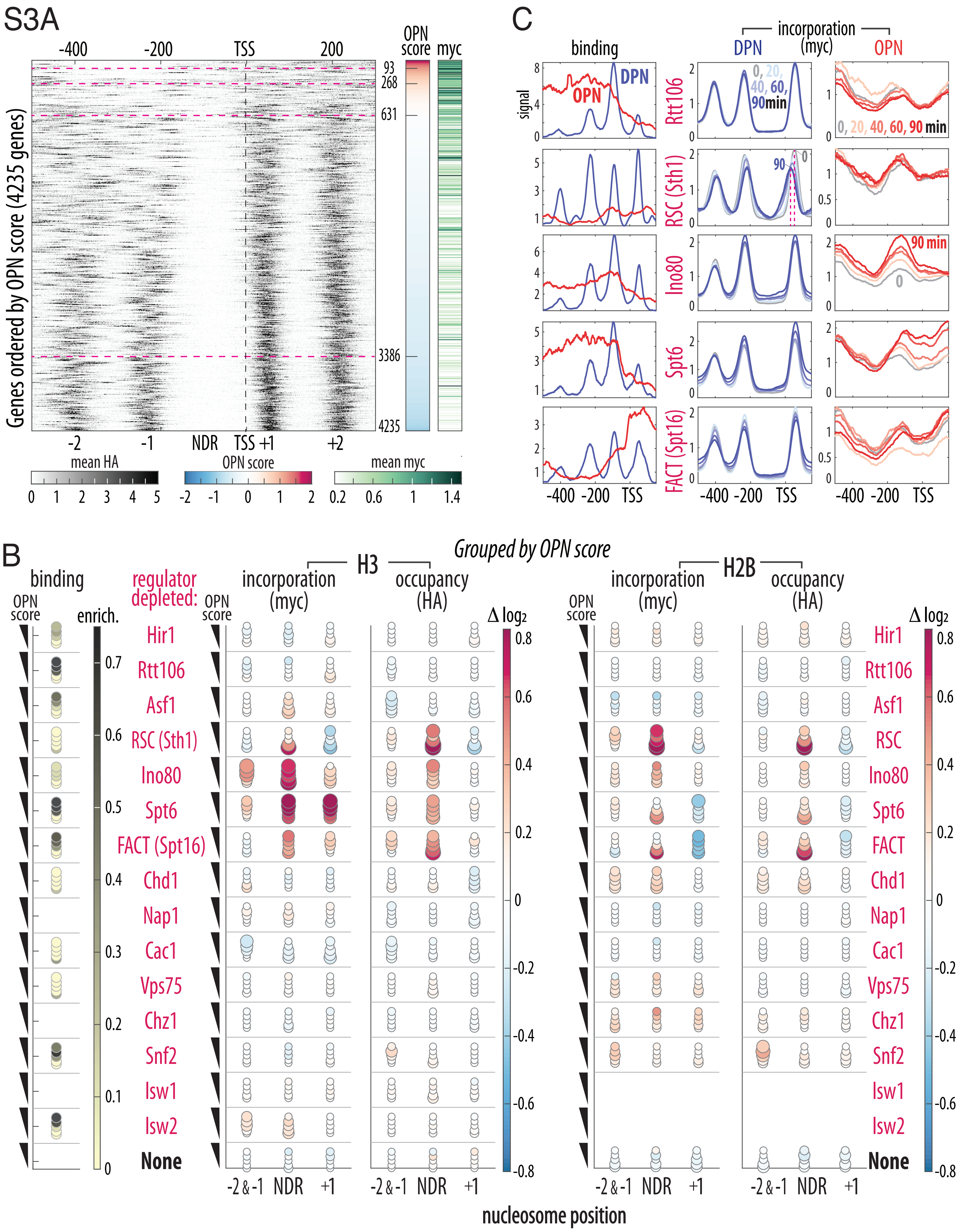

S3A
Genes ordered by OPN score (4235 genes)
-400
-200
TSS
200
-2
-1
NDR
TSS +1
+2
93
268
631
3386
4235
OPN score
myc
mean HA
OPN score
mean myc
C
binding
incorporation (myc)
DPN
OPN
signal
Rtt106
RSC (Sth1)
Ino80
Spt6
FACT (Spt16)
0, 20, 40, 60, 90min
0, 20, 40, 60, 90 min
90 min
-400 -200 TSS
B
Grouped by OPN score
binding
OPN score
enrich.
regulator depleted:
H3
incorporation (myc)
occupancy (HA)
H2B
Δ log2
Hir1
Rtt106
Asf1
RSC (Sth1)
Ino80
Spt6
FACT (Spt16)
Chd1
Nap1
Cac1
Vps75
Chz1
Snf2
Isw1
Isw2
None
RSC
FACT
-2 & -1
NDR
+1
nucleosome position

Figure S3 – Nucleosome exchange and positioning in promoters

A. *Nucleosome organization correlates with histone exchange*. H3 occupancy (HA) across all gene promoters aligned by TSS and ordered by their Occupied Proximal Nucleosome (OPN) score (2)(see Methods). Distance from TSS in bp above, nucleosome position and Nucleosome Depleted Region (NDR) indicated below. H3 incorporation (myc) shown on the right correlates with OPN score (0.47 Pearson).

B. *Effects on promoter histone exchange are observed in only a subset of regulators.* Mean relative change during depletion (log_2) in H3/H2B incorporation/occupancy are shown for the indicated promoter regions corresponding to (A). Relative changes for 5 decreasing OPN score promoter bins (indicated in magenta in (A)) are shown as color, with size of circle corresponding to the p-value of correlation between the signal change and regulator depletion time. Of note, relative changes in NDR are amplified by very low starting nucleosome occupancy. Mean relative enrichment of each regulator in the region surrounding the TSS (-300 bp to +150 bp around the TSS) is shown on left.

C. *Promoter H3 incorporation and position are controlled by specific regulators.* The top 93 OPN (red) and top 849 DPN (Depleted Proximal Promoters having the lowest OPN scores, blue) genes were selected, and the mean myc H3 incorporation signal around their TSS the 5 time points is shown. The steady-state binding profile of the depleted regulator on those same genes is shown on the left. The +1 nucleosome position shift is indicated with dotted lines before (0) and 90 after RSC (Sth1) depletion as reported (3–7).

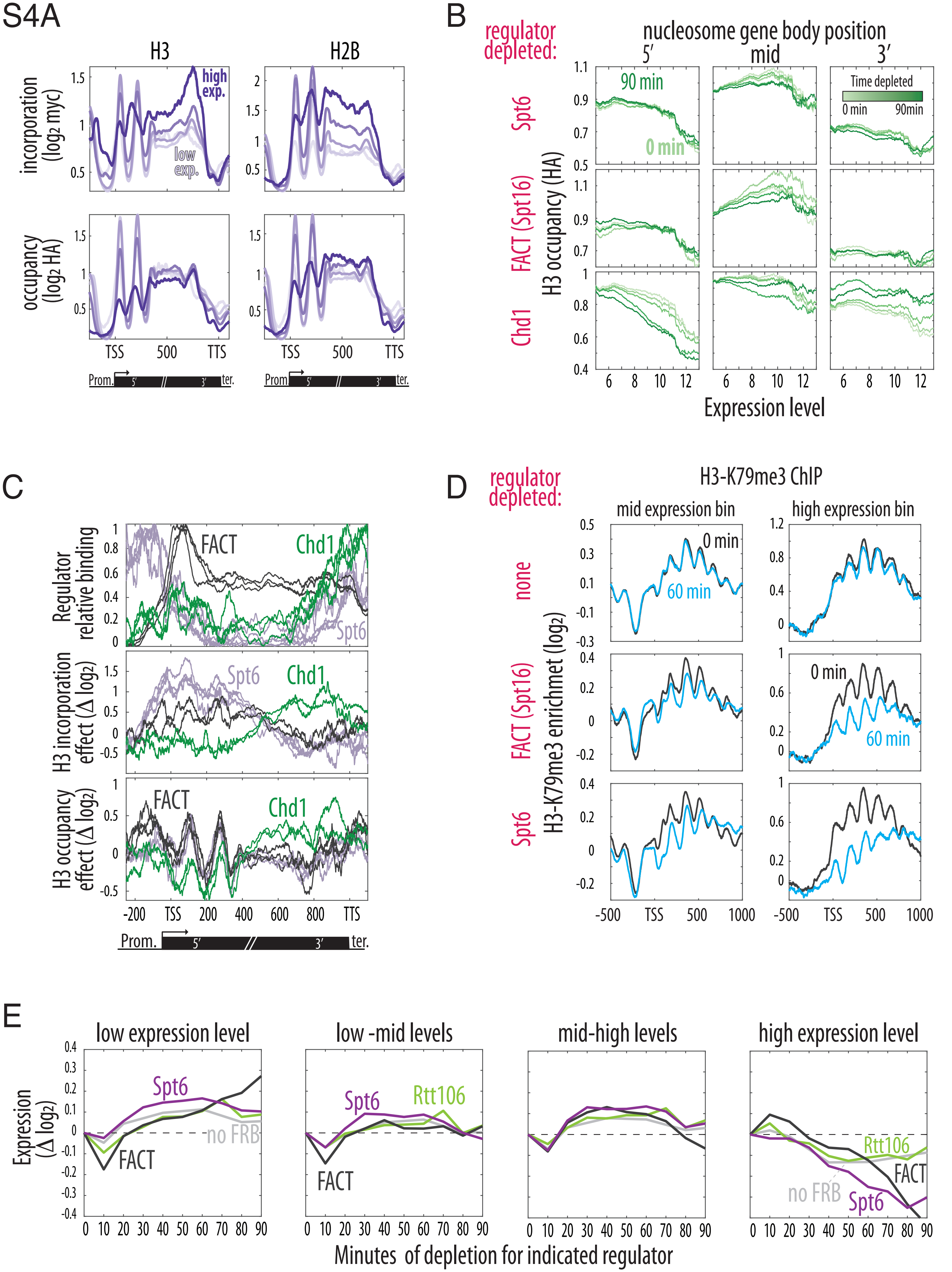
S4A
H3
H2B
incorporation (log2 myc)
occupancy (log2 HA)
high exp.
low exp.
TSS
500
TTS
Prom.
5′
3′
ter.
B
regulator depleted:
nucleosome gene body position
5′
mid
3′
Spt6
FACT (Spt16)
Chd1
H3 occupancy (HA)
90 min
0 min
Time depleted
0 min
90min
Expression level
C
Regulator relative binding
H3 incorporation effect (Δ log2)
H3 occupancy effect (Δ log2)
FACT
Chd1
Spt6
D
regulator depleted:
H3-K79me3 ChIP
mid expression bin
high expression bin
none
FACT (Spt16)
Spt6
H3-K79me3 enrichmet (log2)
0 min
60 min
E
low expression level
low -mid levels
mid-high levels
high expression level
Expression (Δ log2)
Spt6
FACT
no FRB
Rtt106
Minutes of depletion for indicated regulator

Figure S4 – Nucleosome exchange in gene bodies correlates to expression levels

A. *Histone exchange correlates with expression levels*. Genes are divided into 5 expression level bins, and mean H3 and H2B incorporation (myc) and occupancy (HA) during steady state are shown for each group aligned by the TSS and normalized for length. Highest expression corresponds to the darkest shade.

B. Associated with Fig. 2B: HA occupancy levels change with time of regulator depletion across the start, middle and end regions of all gene bodies.

C. Associated with Fig. 2C. Data here is shown as individual repeats.

D. *Histone H3-K79me3 marks are decreased with the depletion of FACT and Spt6.* ChIP-seq using H3-K79me3 antibody on samples before (0 min) and after 1 hour of depletion no regulator (control), Spt16 or Spt6. H3-K79me3 enrichment (log_2) is shown by normalizing each sample to its respective occupancy (HA) levels measured in parallel. Two expression bins are shown, corresponding to middle expression levels (3rd bin in (A)) or the highest expression bin. Note that H3-K79me3 levels are strongly decreased following FACT and Spt6 depletion in an expression-dependent manner, as previously reported (8).

E. Genome-wide mRNA analysis following the depletion of Spt6, FACT, Rtt106, or no FRB control in which no regulator is depleted. Rapamycin was added, samples were taken every 10 minutes, and expression was measured. Log_2 changes in expression relative to pre-depletion were analyzed by looking at the same bins of expression levels in Fig. 2A and elsewhere.

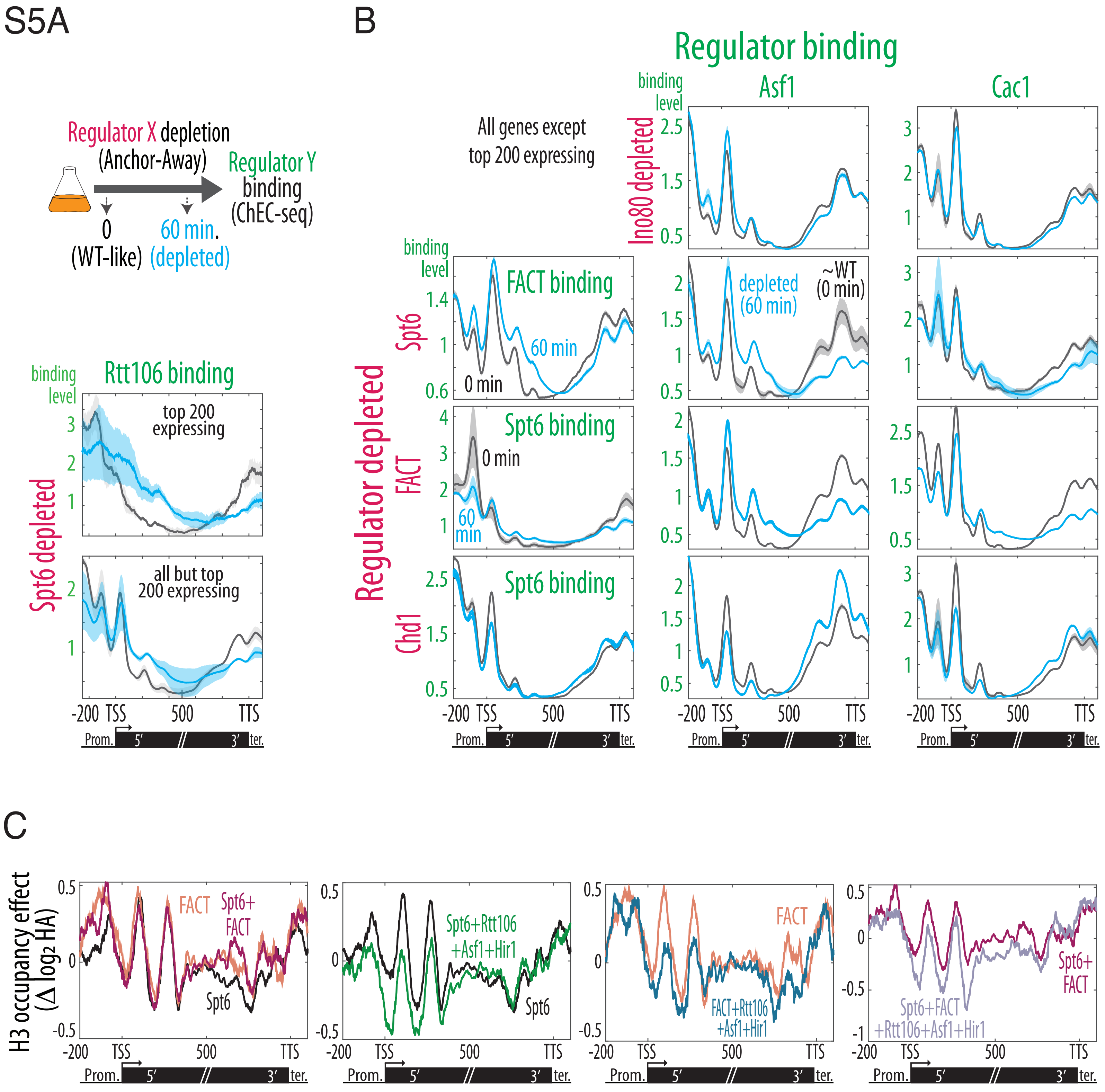

S5A
B
C
Regulator X depletion
(Anchor-Away)
Regulator Y binding (ChEC-seq)
0 (WT-like)
60 min. (depleted)
Rtt106 binding
binding level
top 200 expressing
all but top 200 expressing
Spt6 depleted
Regulator binding
Asf1
Cac1
All genes except top 200 expressing
Ino80 depleted
Regulator depleted
Spt6
FACT
Chd1
FACT binding
Spt6 binding
60 min
0 min
depleted (60 min)
~WT (0 min)
-200 TSS 500 TTS
Prom. 5′ 3′ ter.
H3 occupancy effect (Δ log2 HA)
FACT
Spt6+ FACT
Spt6
Spt6+Rtt106 +Asf1+Hir1
FACT+Rtt106 +Asf1+Hir1
Spt6+FACT +Rtt106+Asf1+Hir1

Figure S5 – Chaperone re-localization and activity following counterpart depletion

A-B. Associated with Fig. 3A: (A) binding profiles for Rtt106 in Spt6-depleted cells as in Fig. 3A for top 200 expressing genes (top) or all remaining genes (bottom). (B) binding for same chaperones and depletions as in Fig 3A for all expression levels except the top 200 highest expressing.

C. Associated with Fig. 4B: HA changes for the indicated co-depletions.

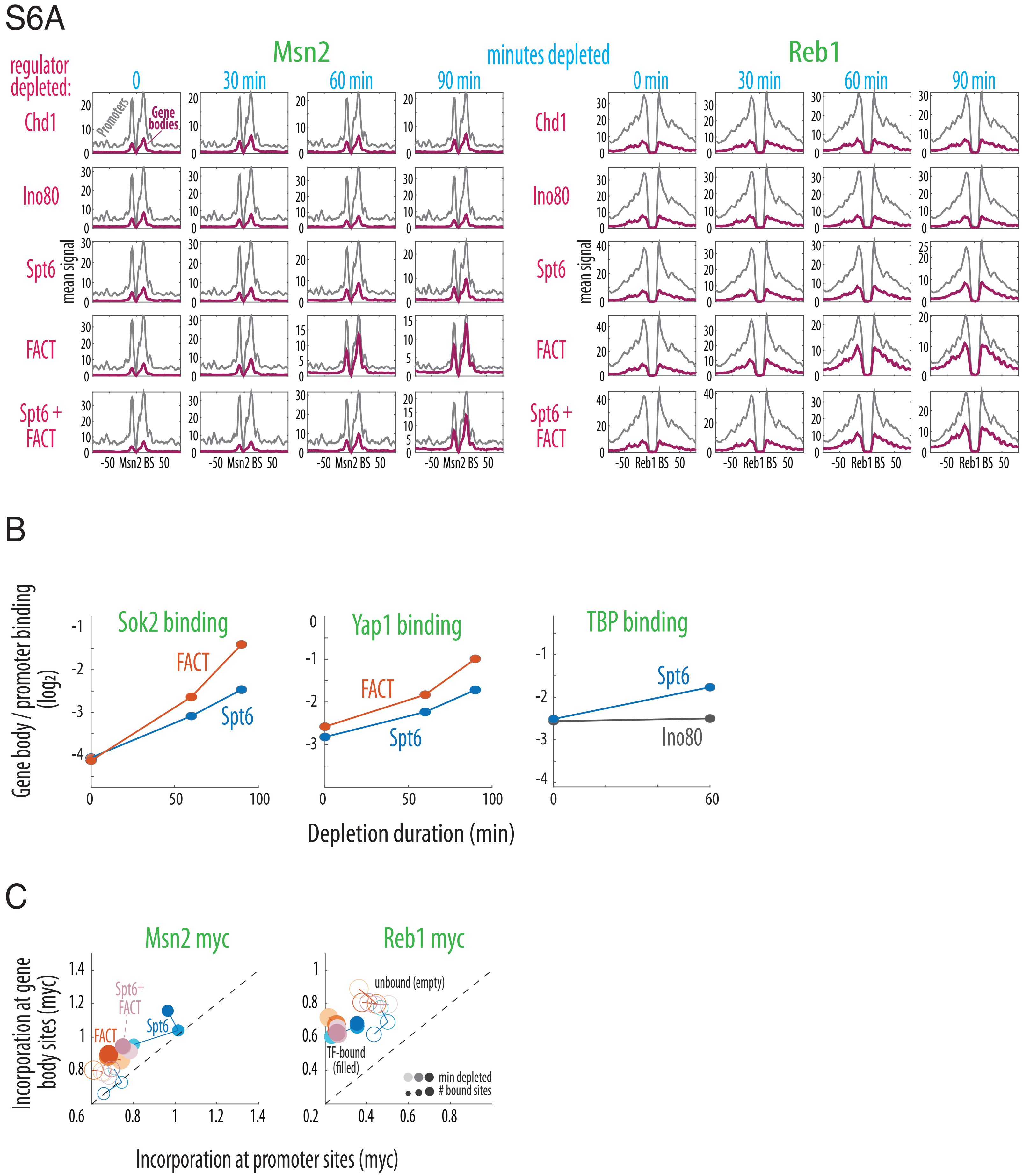
S6A
Msn2
minutes depleted
Reb1
regulator depleted:
0
30 min
60 min
90 min
0 min
Chd1
Ino80
Spt6
FACT
Spt6 + FACT
Promoters
Gene bodies
mean signal
-50 Msn2 BS 50
-50 Reb1 BS 50
B
Sok2 binding
Yap1 binding
TBP binding
FACT
Spt6
Ino80
Gene body / promoter binding (log2)
Depletion duration (min)
C
Msn2 myc
Reb1 myc
Spt6+ FACT
unbound (empty)
TF-bound (filled)
min depleted
bound sites
Incorporation at gene body sites (myc)
Incorporation at promoter sites (myc)

Figure S6 – Spurious gene body binding following depletion of a subset of histone regulators

A. Associated with Fig. 5C: Msn2 (left) and Reb1 gene body binding (purple) vs promoter binding (grey) 0, 30, 60 or 90 minutes following the co/depletion of the indicated regulators. Data is aligned on the respective binding motif, with 614 promoter and 527 gene body motifs for Reb1.

B. Associated with Fig. 5E: Gene body vs promoter binding for Sok2, Yap1 and TBP following the depletion of the indicated regulator.

C. Associated with Fig. 5G: H3 incorporation changes with time at final Msn2 and Reb1 binding sites during the depletion of FACT, Spt6 or both.

References

1. Gelbart,M.E., Rechsteiner,T., Richmond,T.J. and Tsukiyama,T. (2023) Interactions of Isw2 Chromatin Remodeling Complex with Nucleosomal Arrays: Analyses Using Recombinant Yeast Histones and Immobilized Templates. *https://doi.org/10.1128/MCB.21.6.2098-2106.2001*, **21**, 2098–2106.
2. Tirosh,I. and Barkai,N. (2008) Two strategies for gene regulation by promoter nucleosomes. *Genome Res.*, 10.1101/gr.076059.108.
3. Yen,K., Vinayachandran,V. and Pugh,B.F. (2013) SWR-C and INO80 Chromatin Remodelers Recognize Nucleosome-free Regions Near +1 Nucleosomes. *Cell*, **154**, 1246–1256.
4. Klein-Brill,A., Joseph-Strauss,D., Appleboim,A. and Friedman,N. (2019) Dynamics of Chromatin and Transcription during Transient Depletion of the RSC Chromatin Remodeling Complex. *Cell Rep.*, **26**, 279-292.e5.
5. Badis,G., Chan,E.T., van Bakel,H., Pena-Castillo,L., Tillo,D., Tsui,K., Carlson,C.D., Gossett,A.J., Hasinoff,M.J., Warren,C.L., *et al.* (2008) A library of yeast transcription factor motifs reveals a widespread function for Rsc3 in targeting nucleosome exclusion at promoters. *Mol Cell*, **32**, 878–887.
6. Hartley,P.D. and Madhani,H.D. (2009) Mechanisms that specify promoter nucleosome location and identity. *Cell*, **137**, 445–458.
7. Parnell,T.J., Huff,J.T. and Cairns,B.R. (2008) RSC regulates nucleosome positioning at Pol II genes and density at Pol III genes. *Embo j*, **27**, 100–110.
8. Jeronimo,C., Poitras,C. and Robert,F. (2019) Histone Recycling by FACT and Spt6 during Transcription Prevents the Scrambling of Histone Modifications. *Cell Rep.*, **28**.

Table S1 Strains generated and used in this study

All strains are BY4741 MATa his3Δ1 leu2Δ0 LYS2 met15Δ0 ura3Δ0
Additional genetic manipulations are indicated per strain
Modifications without an indicated selection marker were performed with CRISPR
*The tor1-1 point mutation was taken from HHY168 (Haruki H et al, Mol. Cell 2008) and in fact corresponds to the tor2-1 S1971I mutation (Helliwell SB et al, MBoC 2017) inserted in the TOR1 gene.
Abbreviations: AA; Anchor Away, Fast; ENLYFQS TEV cleavage site

Strain name	Genotype	Short description
YGY691	HTB2-GGS-TEV HHT2-GGS-HA-Fast-MYC bar1 , tor1-1*(Ser1971Ile), fpr1 , RPL13A-2xFKB12	**H3 AA base strain**
YGY692	HHT1-GGS-TEV, HTB2-GGS-HA-Fast-MYC, bar1 , tor1-1*(Ser1971Ile), fpr1 , RPL13A-2xFKB12	**H2B AA base strain**
YGY693	HTB2-GGS-TEV HHT2-GGS-HA-Fast-MYC bar1 , tor1-1*(Ser1971Ile), fpr1 , RPL13A-2xFKB12, Spt16-FRB::KANMX	**H3 FACT AA**
YGY694	HHT1-GGS-TEV, HTB2-GGS-HA-Fast-MYC, bar1 , tor1-1*(Ser1971Ile), fpr1 , RPL13A-2xFKB12, Spt16-FRB::KANMX	**H2B FACT AA**
YGY695	HTB2-GGS-TEV HHT2-GGS-HA-Fast-MYC bar1 , tor1-1*(Ser1971Ile), fpr1 , RPL13A-2xFKB12, Spt6-FRB::KANMX	**H3 Spt6 AA**
YGY696	HTB2-GGS-TEV HHT2-GGS-HA-Fast-MYC bar1 , tor1-1*(Ser1971Ile), fpr1 , RPL13A-2xFKB12, Spt6-FRB::KANMX	**H3 Spt6 AA**
YGY697	HHT1-GGS-TEV, HTB2-GGS-HA-Fast-MYC, bar1 , tor1-1*(Ser1971Ile), fpr1 , RPL13A-2xFKB12, Spt6-FRB::KANMX	**H2B Spt6 AA**
YGY698	HHT1-GGS-TEV, HTB2-GGS-HA-Fast-MYC, bar1 , tor1-1*(Ser1971Ile), fpr1 , RPL13A-2xFKB12, Spt6-FRB::KANMX	**H2B Spt6 AA**
YGY699	HTB2-GGS-TEV HHT2-GGS-HA-Fast-MYC bar1 , tor1-1*(Ser1971Ile), fpr1 , RPL13A-2xFKB12, Nap1-FRB::KANMX	**H3 Nap1 AA**
YGY700	HTB2-GGS-TEV HHT2-GGS-HA-Fast-MYC bar1 , tor1-1*(Ser1971Ile), fpr1 , RPL13A-2xFKB12, Chz1-FRB::KANMX	**H3 Chz1 AA**
YGY701	HTB2-GGS-TEV HHT2-GGS-HA-Fast-MYC bar1 , tor1-1*(Ser1971Ile), fpr1 , RPL13A-2xFKB12, Asf1-FRB::KANMX	**H3 Asf1 AA**
YGY702	HTB2-GGS-TEV HHT2-GGS-HA-Fast-MYC bar1 , tor1-1*(Ser1971Ile), fpr1 , RPL13A-2xFKB12, Vps75-FRB::KANMX	**H3 Vps75 AA**
YGY703	HTB2-GGS-TEV HHT2-GGS-HA-Fast-MYC bar1 , tor1-1*(Ser1971Ile), fpr1 , RPL13A-2xFKB12, Cac1-FRB::KANMX	**H3 Cac1 AA**
YGY704	HTB2-GGS-TEV HHT2-GGS-HA-Fast-MYC bar1 , tor1-1*(Ser1971Ile), fpr1 , RPL13A-2xFKB12, Rtt106-FRB::KANMX	**H3 Rtt106 AA**
YGY705	HTB2-GGS-TEV HHT2-GGS-HA-Fast-MYC bar1 , tor1-1*(Ser1971Ile), fpr1 , RPL13A-2xFKB12, Hir1-FRB::KANMX	**H3 Hir1 AA**
YGY706	HHT1-GGS-TEV, HTB2-GGS-HA-Fast-MYC, bar1 , tor1-1*(Ser1971Ile), fpr1 , RPL13A-2xFKB12, Nap1-FRB::KANMX	**H2B Nap1 AA**

YGY707	HHT1-GGS-TEV, HTB2-GGS-HA-Fast-MYC, bar1 , tor1-1*(Ser1971Ile), fpr1 , RPL13A-2xFKB12, Chz1-FRB::KANMX	**H2B Chz1 AA**
YGY708	HHT1-GGS-TEV, HTB2-GGS-HA-Fast-MYC, bar1 , tor1-1*(Ser1971Ile), fpr1 , RPL13A-2xFKB12, Asf1-FRB::KANMX	**H2B Asf1 AA**
YGY709	HHT1-GGS-TEV, HTB2-GGS-HA-Fast-MYC, bar1 , tor1-1*(Ser1971Ile), fpr1 , RPL13A-2xFKB12, Vps75-FRB::KANMX	**H2B Vps75 AA**
YGY710	HHT1-GGS-TEV, HTB2-GGS-HA-Fast-MYC, bar1 , tor1-1*(Ser1971Ile), fpr1 , RPL13A-2xFKB12, Cac1-FRB::KANMX	**H2B Cac1 AA**
YGY711	HHT1-GGS-TEV, HTB2-GGS-HA-Fast-MYC, bar1 , tor1-1*(Ser1971Ile), fpr1 , RPL13A-2xFKB12, Rtt106-FRB::KANMX	**H2B Rtt106 AA**
YGY712	HHT1-GGS-TEV, HTB2-GGS-HA-Fast-MYC, bar1 , tor1-1*(Ser1971Ile), fpr1 , RPL13A-2xFKB12, Hir1-FRB::KANMX	**H2B Hir1 AA**
YGY715	HTB2-GGS-TEV HHT2-GGS-HA-Fast-MYC bar1 , tor1-1*(Ser1971Ile), fpr1 , RPL13A-2xFKB12, Spt16-FRB::KANMX, Msn2-MNase	**FACT AA Msn2-MNase**
YGY716	HTB2-GGS-TEV HHT2-GGS-HA-Fast-MYC bar1 , tor1-1*(Ser1971Ile), fpr1 , RPL13A-2xFKB12, Spt16-FRB::KANMX, Msn2-MNase	**FACT AA Msn2-Mnase**
YGY719	HTB2-GGS-TEV HHT2-GGS-HA-Fast-MYC bar1 , tor1-1*(Ser1971Ile), fpr1 , RPL13A-2xFKB12, Spt16-FRB::KANMX, Sok2-MNase	**FACT AA Sok2-Mnase**
YGY720	HTB2-GGS-TEV HHT2-GGS-HA-Fast-MYC bar1 , tor1-1*(Ser1971Ile), fpr1 , RPL13A-2xFKB12, Spt6-FRB::KANMX, Sok2-MNase	**SPT6 AA Sok2-Mnase**
YGY723	HTB2-GGS-TEV HHT2-GGS-HA-Fast-MYC bar1 , tor1-1*(Ser1971Ile), fpr1 , RPL13A-2xFKB12, Spt6-FRB::KANMX, Msn2-MNase	**SPT6 AA Msn2-Mnase**
YGY726	HTB2-GGS-TEV HHT2-GGS-HA-Fast-MYC bar1 , tor1-1*(Ser1971Ile), fpr1 , RPL13A-2xFKB12, Hir1-FRB::KANMX, Msn2-MNase	**Hir1 AA Msn2-MNase**
YGY729	HTB2-GGS-TEV HHT2-GGS-HA-Fast-MYC bar1 , tor1-1*(Ser1971Ile), fpr1 , RPL13A-2xFKB12, Hir1-FRB::KANMX, sok2-MNase	**Hir1 AA sok2-MNase**
YGY734	HTB2-GGS-TEV HHT2-GGS-HA-Fast-MYC bar1 , tor1-1*(Ser1971Ile), fpr1 , RPL13A-2xFKB12, Spt16-FRB::KANMX, Reb1-Nter-MNase	**FACT AA MNase-Reb1**
YGY735	HTB2-GGS-TEV HHT2-GGS-HA-Fast-MYC bar1 , tor1-1*(Ser1971Ile), fpr1 , RPL13A-2xFKB12, Spt6-FRB::KANMX, Reb1-Nter-MNase	**Spt6 AA MNase-Reb1**
YGY737	HTB2-GGS-TEV HHT2-GGS-HA-Fast-MYC bar1 , tor1-1*(Ser1971Ile), fpr1 , RPL13A-2xFKB12, Hir1-FRB::KANMX, Reb1-Nter-MNase	**Hir1 AA MNase-Reb1**
YGY738	HTB2-GGS-TEV HHT2-GGS-HA-Fast-MYC bar1 , tor1-1*(Ser1971Ile), fpr1 , RPL13A-2xFKB12, Spt6-FRB & Spt16-FRB double	**H3 Spt6&FACT AA**
YGY739	HTB2-GGS-TEV HHT2-GGS-HA-Fast-MYC bar1 , tor1-1*(Ser1971Ile), fpr1 , RPL13A-2xFKB12, Spt6-FRB & Spt16-FRB double	**H3 Spt6&FACT AA**
YGY740	HTB2-GGS-TEV HHT2-GGS-HA-Fast-MYC bar1 , tor1-1*(Ser1971Ile), fpr1 , RPL13A-2xFKB12, Hir1-FRB, Rtt106 & Asf1-FRB triple	**H3 Hir1, Rtt106 & Asf1 AA**
YGY741	HTB2-GGS-TEV HHT2-GGS-HA-Fast-MYC bar1 , tor1-1*(Ser1971Ile), fpr1 , RPL13A-2xFKB12, Hir1-FRB, Rtt106 & Asf1-FRB triple , spt6-FRB	**H3 Spt6, Hir1, Rtt106 & Asf1 AA**
YGY742	HTB2-GGS-TEV HHT2-GGS-HA-Fast-MYC bar1 , tor1-1*(Ser1971Ile), fpr1 , RPL13A-2xFKB12, Hir1-FRB, Rtt106 & Asf1-FRB triple, spt16-FRB	**H3 FACT, Hir1, Rtt106 & Asf1 AA**
YGY743	HTB2-GGS-TEV HHT2-GGS-HA-Fast-MYC bar1 , tor1-1*(Ser1971Ile), fpr1 , RPL13A-2xFKB12, Hir1-FRB, Rtt106 & Asf1-FRB triple, spt16-FRB	**H3 FACT, Hir1, Rtt106 & Asf1 AA**

YGY745	HTB2-GGS-TEV HHT2-GGS-HA-Fast-MYC bar1 , tor1-1*(Ser1971Ile), fpr1 , RPL13A-2xFKB12, Hir1-FRB, Rtt106 & Asf1-FRB triple , Spt6-FRB & spt16-FRB double	**H3 Spt6, FACT, Hir1, Rtt106 & Asf1 AA**
YGY746	HTB2-GGS-TEV HHT2-GGS-HA-Fast-MYC bar1 , tor1-1*(Ser1971Ile), fpr1 , RPL13A-2xFKB12, Sth1-FRB::KANMX	**H3 RSC AA**
YGY747	HTB2-GGS-TEV HHT2-GGS-HA-Fast-MYC bar1 , tor1-1*(Ser1971Ile), fpr1 , RPL13A-2xFKB12, Sth1-FRB::KANMX	**H3 RSC AA**
YGY748	HTB2-GGS-TEV HHT2-GGS-HA-Fast-MYC bar1 , tor1-1*(Ser1971Ile), fpr1 , RPL13A-2xFKB12, Snf2-FRB::KANMX	**H3 SWI/SNF AA**
YGY749	HTB2-GGS-TEV HHT2-GGS-HA-Fast-MYC bar1 , tor1-1*(Ser1971Ile), fpr1 , RPL13A-2xFKB12, Snf2-FRB::KANMX	**H3 SWI/SNF AA**
YGY750	HTB2-GGS-TEV HHT2-GGS-HA-Fast-MYC bar1 , tor1-1*(Ser1971Ile), fpr1 , RPL13A-2xFKB12, Ino80-FRB::KANMX	**H3 Ino80 AA**
YGY751	HHT1-GGS-TEV, HTB2-GGS-HA-Fast-MYC, bar1 , tor1-1*(Ser1971Ile), fpr1 , RPL13A-2xFKB12, Sth1-FRB::KANMX	**H2B RSC AA**
YGY752	HHT1-GGS-TEV, HTB2-GGS-HA-Fast-MYC, bar1 , tor1-1*(Ser1971Ile), fpr1 , RPL13A-2xFKB12, Snf2-FRB::KANMX	**H2B SWI/SNF AA**
YGY753	HHT1-GGS-TEV, HTB2-GGS-HA-Fast-MYC, bar1 , tor1-1*(Ser1971Ile), fpr1 , RPL13A-2xFKB12, Ino80-FRB::KANMX	**H2B Ino80 AA**
YGY754	HTB2-GGS-TEV HHT2-GGS-HA-Fast-MYC bar1 , tor1-1*(Ser1971Ile), fpr1 , RPL13A-2xFKB12, Chd1-FRB::KANMX	**H3 Chd1 AA**
YGY755	HTB2-GGS-TEV HHT2-GGS-HA-Fast-MYC bar1 , tor1-1*(Ser1971Ile), fpr1 , RPL13A-2xFKB12, ISW1-FRB::KANMX	**H3 ISW-1 AA**
YGY756	HTB2-GGS-TEV HHT2-GGS-HA-Fast-MYC bar1 , tor1-1*(Ser1971Ile), fpr1 , RPL13A-2xFKB12, ISW2-FRB::KANMX	**H3 ISW-2 AA**
YGY757	HTB2-GGS-TEV HHT2-GGS-HA-Fast-MYC bar1 , tor1-1*(Ser1971Ile), fpr1 , RPL13A-2xFKB12, Spt6-FRB & Spt16-FRB double , Msn2-MNase	**Spt6&FACT AA Msn2-Mnase**
YGY758	HTB2-GGS-TEV HHT2-GGS-HA-Fast-MYC bar1 , tor1-1*(Ser1971Ile), fpr1 , RPL13A-2xFKB12, Spt6-FRB & Spt16-FRB double , Msn2-MNase	**Spt6&FACT AA Msn2-MNase**
YGY759	HHT1-GGS-TEV, HTB2-GGS-HA-Fast-MYC, bar1 , tor1-1*(Ser1971Ile), fpr1 , RPL13A-2xFKB12, Ino80-FRB::KANMX, Msn2-MNase	**Ino80 AA Msn2-Mnase**
YGY760	HTB2-GGS-TEV HHT2-GGS-HA-Fast-MYC bar1 , tor1-1*(Ser1971Ile), fpr1 , RPL13A-2xFKB12, Chd1-FRB::KANMX, Msn2-MNase	**Chd1 AA Msn2-MNase**
YGY761	HTB2-GGS-TEV HHT2-GGS-HA-Fast-MYC bar1 , tor1-1*(Ser1971Ile), fpr1 , RPL13A-2xFKB12, Spt6-FRB & Spt16-FRB double , Nter MNase-Reb1	**Spt6&FACT AA MNase-Reb1**
YGY762	HHT1-GGS-TEV, HTB2-GGS-HA-Fast-MYC, bar1 , tor1-1*(Ser1971Ile), fpr1 , RPL13A-2xFKB12, Ino80-FRB::KANMX, Nter MNase-Reb1	**Ino80 AA MNase-Reb1**
YGY763	HHT1-GGS-TEV, HTB2-GGS-HA-Fast-MYC, bar1 , tor1-1*(Ser1971Ile), fpr1 , RPL13A-2xFKB12, Ino80-FRB::KANMX, Nter MNase-Reb1	**Ino80 AA MNase-Reb1**
YGY764	HTB2-GGS-TEV HHT2-GGS-HA-Fast-MYC bar1 , tor1-1*(Ser1971Ile), fpr1 , RPL13A-2xFKB12, Chd1-FRB::KANMX, Nter MNase-Reb1	**Chd1 AA MNase-Reb1**
YGY776	Spt6-MNase-KANMX	**Spt6-Mnase**
YGY777	Spt16FACT-MNase-KANMX	**FACT-Mnase**
YGY778	Ino80-MNase-KANMX	**Ino80-Mnase**

YGY779	Sth1-MNase-KANMX	**Sth1-Mnase**
YGY780	Chd1-MNase-KANMX	**Chd1-Mnase**
YGY781	Asf1-MNase-KANMX	**Asf1-Mnase**
YGY782	Asf1-MNase-KANMX	**Asf1-Mnase**
YGY783	Iswi2-MNase-KANMX	**Iswi2-Mnase**
YGY784	Rtt106-MNase-KANMX	**Rtt106-Mnase**
YGY785	Vps75-MNase-KANMX	**Vps75-Mnase**
YGY787	Nap1-MNase-KANMX	**Nap1-Mnase**
YGY788	Nap1-MNase-KANMX	**Nap1-Mnase**
YGY789	Rlf2-MNase-KANMX	**Rlf2-Mnase**
YGY790	Snf2-MNase-KANMX	**Snf2-Mnase**
YGY791	Hir1-MNase-KANMX	**Hir1-Mnase**
YGY792	HTB2-GGS-TEV HHT2-GGS-HA-Fast-MYC bar1 , tor1-1*(Ser1971Ile), fpr1 , RPL13A-2xFKB12, Spt6-FRB::KANMX, Spt16-MNase (insert has KANMX)	**Spt6 AA FACT-MNase**
YGY793	HTB2-GGS-TEV HHT2-GGS-HA-Fast-MYC bar1 , tor1-1*(Ser1971Ile), fpr1 , RPL13A-2xFKB12, Spt6-FRB::KANMX, Spt16-MNase (insert has KANMX)	**Spt6 AA FACT-Mnase**
YGY795	HTB2-GGS-TEV HHT2-GGS-HA-Fast-MYC bar1 , tor1-1*(Ser1971Ile), fpr1 , RPL13A-2xFKB12, Spt6-FRB::KANMX, Asf1-MNase (insert has KANMX)	**Spt6 AA Asf1-Mnase**
YGY796	HTB2-GGS-TEV HHT2-GGS-HA-Fast-MYC bar1 , tor1-1*(Ser1971Ile), fpr1 , RPL13A-2xFKB12, Spt6-FRB::KANMX, Asf1-MNase (insert has KANMX)	**Spt6 AA Asf1-Mnase**
YGY801	HTB2-GGS-TEV HHT2-GGS-HA-Fast-MYC bar1 , tor1-1*(Ser1971Ile), fpr1 , RPL13A-2xFKB12, Spt6-FRB::KANMX, Cac1-MNase (insert has KANMX)	**Spt6 AA Cac1-MNase**
YGY802	HTB2-GGS-TEV HHT2-GGS-HA-Fast-MYC bar1 , tor1-1*(Ser1971Ile), fpr1 , RPL13A-2xFKB12, Spt6-FRB::KANMX, Cac1-MNase (insert has KANMX)	**Spt6 AA Cac1-Mnase**
YGY804	HTB2-GGS-TEV HHT2-GGS-HA-Fast-MYC bar1 , tor1-1*(Ser1971Ile), fpr1 , RPL13A-2xFKB12, FACT-FRB::KANMX, Asf1-MNase (insert has KANMX)	**FACT AA Asf1-MNase**
YGY805	HTB2-GGS-TEV HHT2-GGS-HA-Fast-MYC bar1 , tor1-1*(Ser1971Ile), fpr1 , RPL13A-2xFKB12, FACT-FRB::KANMX, Cac1-MNase (insert has KANMX)	**FACT AA Cac1-Mnase**
YGY806	HTB2-GGS-TEV HHT2-GGS-HA-Fast-MYC bar1 , tor1-1*(Ser1971Ile), fpr1 , RPL13A-2xFKB12, Chd1-FRB::KANMX, Asf1-MNase (insert has KANMX)	**Chd1 AA Asf1-MNase**
YGY807	HTB2-GGS-TEV HHT2-GGS-HA-Fast-MYC bar1 , tor1-1*(Ser1971Ile), fpr1 , RPL13A-2xFKB12, Chd1-FRB::KANMX, Cac1-MNase (insert has KANMX)	**Chd1 AA Cac1-MNase**
YGY808	HTB2-GGS-TEV HHT2-GGS-HA-Fast-MYC bar1 , tor1-1*(Ser1971Ile), fpr1 , RPL13A-2xFKB12, Chd1-FRB::KANMX, Spt16-MNase (insert has KANMX)	**Chd1 AA FACT-MNase**

YGY809	HTB2-GGS-TEV HHT2-GGS-HA-Fast-MYC bar1 , tor1-1*(Ser1971Ile), fpr1 , RPL13A-2xFKB12, Ino80-FRB::KANMX, Asf1-MNase (insert has KANMX)	**Ino80 AA Asf1-MNase**
YGY810	HTB2-GGS-TEV HHT2-GGS-HA-Fast-MYC bar1 , tor1-1*(Ser1971Ile), fpr1 , RPL13A-2xFKB12, Ino80-FRB::KANMX, Cac1-MNase (insert has KANMX)	**Ino80 AA Cac1-MNase**
YGY811	HTB2-GGS-TEV HHT2-GGS-HA-Fast-MYC bar1 , tor1-1*(Ser1971Ile), fpr1 , RPL13A-2xFKB12, Spt16-FRB::KANMX, Cac1-FRB (insert has KANMX)	**H3 FACT & Cac1 AA**
YGY812	HTB2-GGS-TEV HHT2-GGS-HA-Fast-MYC bar1 , tor1-1*(Ser1971Ile), fpr1 , RPL13A-2xFKB12, Spt6-FRB::KANMX, Cac1-FRB (insert has KANMX)	**H3 Spt6 & Cac1 AA**
YGY813	HHT1-GGS-TEV, HTB2-GGS-HA-Fast-MYC, bar1 , tor1-1*(Ser1971Ile), fpr1 , RPL13A-2xFKB12, Chd1-FRB::KANMX	**H2B Chd1 AA**
YGY814	HTB2-GGS-TEV HHT2-GGS-HA-Fast-MYC bar1 , tor1-1*(Ser1971Ile), fpr1 , RPL13A-2xFKB12, Spt16-FRB::KANMX, SPT6-MNase (insert has KANMX)	**FACT AA Spt6-Mnase**
YGY815	HTB2-GGS-TEV HHT2-GGS-HA-Fast-MYC bar1 , tor1-1*(Ser1971Ile), fpr1 , RPL13A-2xFKB12, Spt16-FRB::KANMX, SPT6-MNase (insert has KANMX)	**FACT AA Spt6-Mnase**
YGY816	HTB2-GGS-TEV HHT2-GGS-HA-Fast-MYC bar1 , tor1-1*(Ser1971Ile), fpr1 , RPL13A-2xFKB12, Chd1-FRB::KANMX, SPT6-MNase (insert has KANMX)	**Chd1 AA Spt6-Mnase**
YGY817	HTB2-GGS-TEV HHT2-GGS-HA-Fast-MYC bar1 , tor1-1*(Ser1971Ile), fpr1 , RPL13A-2xFKB12, Chd1-FRB::KANMX, SPT6-MNase (insert has KANMX)	**Chd1 AA Spt6-Mnase**
YGY818	HTB2-GGS-TEV HHT2-GGS-HA-Fast-MYC bar1 , tor1-1*(Ser1971Ile), fpr1 , RPL13A-2xFKB12, Spt6-FRB & Spt16-FRB double , Asf1-MNase (insert has KANMX)	**Spt6&FACT AA Asf1-Mnase**
YGY819	HTB2-GGS-TEV HHT2-GGS-HA-Fast-MYC bar1 , tor1-1*(Ser1971Ile), fpr1 , RPL13A-2xFKB12, Spt6-FRB & Spt16-FRB double , Asf1-MNase (insert has KANMX)	**Spt6&FACT AA Asf1-Mnase**
YGY820	HTB2-GGS-TEV HHT2-GGS-HA-Fast-MYC bar1 , tor1-1*(Ser1971Ile), fpr1 , RPL13A-2xFKB12, Spt6-FRB & Spt16-FRB double , Cac1-FRB	**H3 Spt6,FACT&Cac1 AA**
YGY821	HTB2-GGS-TEV HHT2-GGS-HA-Fast-MYC bar1 , tor1-1*(Ser1971Ile), fpr1 , RPL13A-2xFKB12, Spt6-FRB & Spt16-FRB double , cac1-MNase (insert has KANMX)	**Spt6&FACT AA Cac1-Mnase**
YGY826	HTB2-GGS-TEV HHT2-GGS-HA-Fast-MYC bar1 , tor1-1*(Ser1971Ile), fpr1 , RPL13A-2xFKB12, Spt6-FRB::KANMX, Spt15-MNase (insert has KANMX)	**Spt6 AA TBP-Mnase**
YGY831	HTB2-GGS-TEV HHT2-GGS-HA-Fast-MYC bar1 , tor1-1*(Ser1971Ile), fpr1 , RPL13A-2xFKB12, Ino80-FRB::KANMX, Spt15-MNase (insert has KANMX)	**Ino80 AA TBP-MNase**

Table S2. Number of repeats for all experiments

ChIP experiments
Sample nomenclature: Antibody (HA/myc) - sensor (h2b/hht2) - regulator/s depleted (aa)

		Minutes following Rapamycin addition						
**Sample**	**Time 0**	**20**	**30**	**40**	**60**	**90**	**120**	**180**
ha-hht2-aaasf1	1	1	0	1	1	1	1	0
ha-hht2-aacac1	1	1	0	1	1	1	1	0
ha-hht2-aachd1	2	2	0	2	2	2	0	0
ha-hht2-aachz1	1	1	0	1	1	1	1	0
ha-hht2-aahir1	1	1	0	1	1	1	1	0
ha-hht2-aaino80	2	2	0	2	2	2	0	0
ha-hht2-aaisw1	1	1	0	1	1	1	0	0
ha-hht2-aaisw2	1	1	0	1	1	1	0	0
ha-hht2-aanap1	1	1	0	1	1	1	1	0
ha-hht2-aartt106	1	1	0	1	1	1	1	0
ha-hht2-aasnf2	1	1	0	1	1	1	0	0
ha-hht2-aaspt16	4	1	1	2	3	2	3	1
ha-hht2-aaspt6	5	3	0	4	4	4	3	0
ha-hht2-aasth1	1	1	0	1	1	1	0	0
ha-hht2-aavps75	1	1	0	1	1	1	1	0
ha-hht2-aanofrb	2	0	0	0	1	0	1	0
ha-hht2-aaspt6spt16	2	2	0	2	2	2	0	0
ha-hht2-aahir1asf1rtt106	1	1	0	1	1	1	0	0
ha-hht2-aahir1asf1rtt106spt16	1	1	0	1	1	1	0	0
ha-hht2-aahir1asf1rtt106spt6	3	3	0	3	3	3	0	0
ha-hht2-aahir1asf1rtt106spt6spt16	1	1	0	1	1	1	0	0
ha-hht2-aaspt16cac1	1	1	0	1	1	1	0	0
ha-hht2-aaspt6cac1	1	1	0	1	1	1	0	0
ha-hht2-aaspt16spt6cac1	1	1	0	1	1	1	0	0
myc-hht2-aaasf1	1	1	0	1	1	1	1	0
myc-hht2-aacac1	1	1	0	1	1	1	1	0
myc-hht2-aachd1	2	2	0	2	2	2	0	0
myc-hht2-aachz1	1	1	0	1	0	1	1	0
myc-hht2-aahir1	1	1	0	1	1	1	1	0
myc-hht2-aaino80	2	2	0	2	2	2	0	0
myc-hht2-aaisw1	1	1	0	1	1	1	0	0
myc-hht2-aaisw2	1	1	0	1	1	1	0	0
myc-hht2-aanap1	1	1	0	1	1	1	1	0
myc-hht2-aartt106	1	1	0	1	1	1	1	0
myc-hht2-aasnf2	1	1	0	1	1	1	0	0
myc-hht2-aaspt16	5	1	1	2	3	2	3	1
myc-hht2-aaspt6	7	3	0	3	4	4	3	0
myc-hht2-aasth1	1	1	0	1	1	1	0	0
myc-hht2-aavps75	1	1	0	1	1	1	1	0
myc-hht2-aanofrb	2	0	0	0	1	0	1	0
myc-hht2-aaspt6spt16	2	2	0	2	2	2	0	0

myc-hht2-aahir1asf1rtt106	1	1	0	1	1	1	0	0
myc-hht2-aahir1asf1rtt106spt16	1	1	0	1	1	1	0	0
myc-hht2-aahir1asf1rtt106spt6	3	3	0	3	3	3	0	0
myc-hht2-aahir1asf1rtt106spt6spt1	1	1	0	1	1	1	0	0
myc-hht2-aaspt16cac1	1	1	0	1	1	1	0	0
myc-hht2-aaspt6cac1	1	1	0	1	1	1	0	0
myc-hht2-aaspt16spt6cac1	1	1	0	1	1	1	0	0
ha-h2b-aaasf1	1	1	0	1	1	1	1	0
ha-h2b-aacac1	1	1	0	1	1	1	1	0
ha-h2b-aachd1	1	1	0	1	1	1	0	0
ha-h2b-aachz1	1	1	0	1	1	1	1	0
ha-h2b-aahir1	1	1	0	1	1	1	1	0
ha-h2b-aaino80	1	1	0	1	1	1	0	0
ha-h2b-aanap1	1	0	0	1	1	1	1	0
ha-h2b-aartt106	1	1	0	1	1	1	1	0
ha-h2b-aasnf2	1	1	0	1	1	1	0	0
ha-h2b-aaspt16	4	1	1	2	2	2	2	1
ha-h2b-aaspt6	3	1	0	2	2	2	2	1
ha-h2b-aasth1	1	1	0	1	1	1	0	0
ha-h2b-aavps75	1	1	0	1	1	1	1	0
ha-h2b-aanofrb	1	0	0	0	0	0	1	0
myc-h2b-aaasf1	1	1	0	1	1	1	1	0
myc-h2b-aacac1	1	1	0	1	1	1	1	0
myc-h2b-aachd1	1	1	0	1	1	1	0	0
myc-h2b-aachz1	1	1	0	1	1	1	1	0
myc-h2b-aahir1	1	1	0	1	1	1	1	0
myc-h2b-aaino80	1	1	0	1	1	1	0	0
myc-h2b-aanap1	1	1	0	1	1	1	1	0
myc-h2b-aartt106	1	1	0	1	1	1	1	0
myc-h2b-aasnf2	1	1	0	1	1	1	0	0
myc-h2b-aaspt16	4	1	1	2	2	2	2	1
myc-h2b-aasth1	1	1	0	1	1	1	0	0
myc-h2b-aavps75	1	1	0	1	1	1	1	0
myc-h2b-aanofrb	1	0	0	0	0	0	1	0

ChEC TF experiments

Sample nomenclature: Pure/spike (for Fig 5D) - TF measured - regulator/s depleted (aa)

		Minutes following			
**Sample**	**Time 0**	**30**	**60**	**90**	**120**
pure-msn2-aaino80	2	2	2	2	0
pure-msn2-aachd1	2	2	2	2	0
pure-msn2-aaspt16	1	1	1	1	0
pure-msn2-aaspt6	1	1	1	1	0
pure-msn2-aaspt6spt16	2	2	2	2	0
pure-reb1-aaino80	2	2	2	2	0
pure-reb1-aachd1	1	1	1	1	0
pure-reb1-aaspt16	1	1	1	1	0
pure-reb1-aaspt6	2	2	2	2	0
pure-reb1-aaspt6spt16	2	2	2	2	0
pure-sok2-aaspt16	1	0	1	1	1
pure-sok2-aaspt6	1	0	1	1	1
pure-tbp-aaino80	4	0	4	0	0
pure-tbp-aaspt6	4	0	4	0	0
pure-yap1-aaspt16	1	0	1	1	1
pure-yap1-aaspt6	1	0	1	1	1
spike-msn2-aahir1	7	2	7	7	5
spike-msn2-aaspt16	5	2	5	5	3
spike-msn2-aaspt6	3	2	3	3	1
spike-reb1-aahir1	2	2	2	2	0
spike-reb1-aaspt16	2	2	2	2	0
spike-sok2-aahir1	3	0	3	3	3

ChEC regulator experiments

Sample nomenclature: regulator measured - regulator/s depleted (aa)

Sample	Time 0	60
asf1-aachd1	2	2
asf1-aaino80	2	2
asf1-aaspt16	5	3
asf1-aaspt6	2	2
asf1-aaspt6spt16	2	2
cac1-aachd1	2	2
cac1-aaino80	2	1
cac1-aaspt16	1	1
cac1-aaspt6	3	2
cac1-aaspt6spt16	2	2
rtt106-aaspt6	2	3
spt16-aachd1	3	2
spt16-aaspt6	7	7
spt6-aachd1	2	2
spt6-aaspt16	3	3
