# Supplementary material for: Nucleosome retention by histone chaperones and remodelers occludes pervasive DNA–protein binding
Source: Nucleic Acids Res. 2023 Jul 26;51(16):8496–513. doi: 10.1093/nar/gkad615 (PMC10484674; doi:10.1093/nar/gkad615)
Supplement: gkad615_Supplemental_Files [file gkad615_supplemental_files.zip › Supplementary Note 20230607.pdf]

Supplementary Note

In this note, we specifically analyze occupancy measurements using the exchange sensor system and the anchor away strains. As discussed in our manuscript, we did not detect large changes in nucleosome occupancy (HA) following the depletion of most chromatin regulators, despite significant changes observed in histone exchange dynamics (myc). This contrasts reports that measure occupancy changes of tens of percent following the deactivation of Spt6 or FACT. We note that these results are not critical for our main conclusions which concern histone exchange more than occupancy. However, as there is a discrepancy between our data and previous literature, we specifically analyze this point in depth. We summarize analyses and experiments that evaluate the sensor's ability and limits to measure occupancy.

1. We note that for Chd1, we did in fact detect significant changes (30-40%) in occupancy, as shown in Fig 2A. Occupancy changes provoked by Chd1 depletion mirrored the incorporation (myc) changes in both the affected gene regions and the expression level, increasing at the 3' of coding regions in highly expressed genes. These results are highly reproducible across repeats (see Supp Fig S4C). We are therefore able to detect occupancy changes but rather do not see such changes for Spt6 and FACT.

2. We wished to directly test whether the presence of myc in our sensor strains may affect our HA occupancy measurements. We have previously performed ChIP on our sensor using an H3 antibody (Abcam1791) and compared it to parallel HA ChIPs (see Fig S5B in (1)). Reanalyzing this data, we observe the expected high correlation (Fig N1a), demonstrating the reliability of HA measures on histone occupancy.

Next, focusing on HA and H3 ChIP levels as a function of myc levels, we found a potential systematic, yet relatively small, impact of myc levels on HA versus H3 antibody measures. According to this, a two-fold change in myc levels may result in a 9% (2^0.12 $\log_2$) overestimation of occupancy with HA measurement as compared to H3 ChIPs (see Fig N1b). We conclude that the myc tag may somehow increase HA ChIP efficiency slightly through a mechanism we do not understand. We note however that an increase of ~9% cannot explain the lack of HA changes with regulator depletion as compared to previous reports of tens of percent occupancy changes.

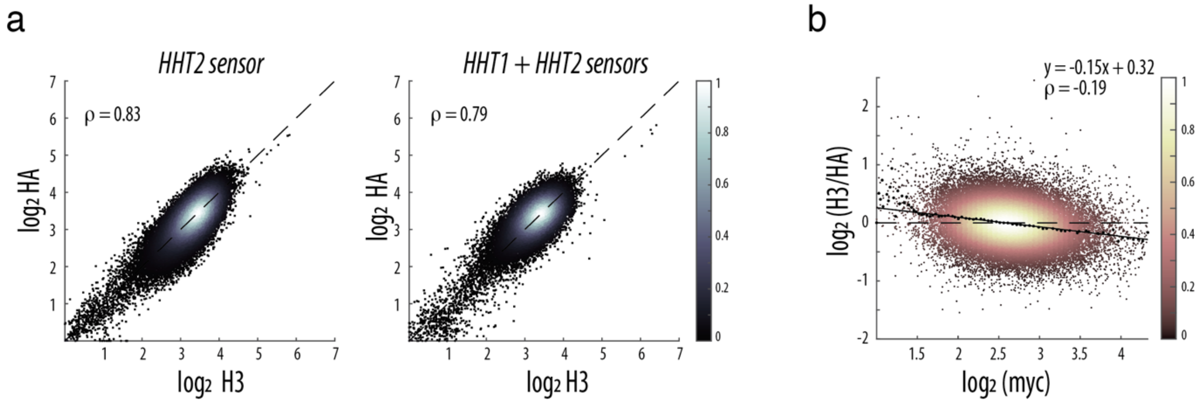

Figure N1. HA occupancy ChIP measurements correlate highly with H3 ChIP, with myc tag slightly increasing HA ChIP efficiency. ChIP was carried out using antibodies directed against H3 alongside the HA and myc sensor epitopes. **(a)** Correlation (Pearson) of the HA ChIPs to their H3 counterparts across all nucleosomes (~60k) in strains with the sensor fused to Hht2 (as are the strains in this study) or both H3 alleles, such that all nucleosomes are necessarily tagged. **(b)** A density plot of all nucleosomes (dots) according to the ratio of H3 to HA levels ($\log_2$) as a function of their myc ChIP values ($\log_2$) is shown. Black dots correspond to mean levels, with a linear fit, its slope, and Pearson correlation

3. We have previously followed histone exchange in cells exposed to oxidative stress, in which we detected occupancy decreasing specifically at Yap1-dependent target promoters and +1,+2 nucleosomes of induced genes (Fig 4 and S4 in (2)). To directly test our ability to detect occupancy changes in Spt6 and FACT anchor-away strains, we repeated this experimental design. We grew cells, depleted the given regulator for one hour, exposed them to H_2O_2, and measured occupancy changes with time (Fig N2). We detect a notable decrease in occupancy specifically at the binding sites of Yap1, an oxidative stress-specific TF, as well as the corresponding target gene promoters (~50%) and +1 & +2 nucleosomes, in line with our previous findings. These results suggest that our HA ChIP measurements can detect occupancy in the previous and the current sensor strains and experimental setups.

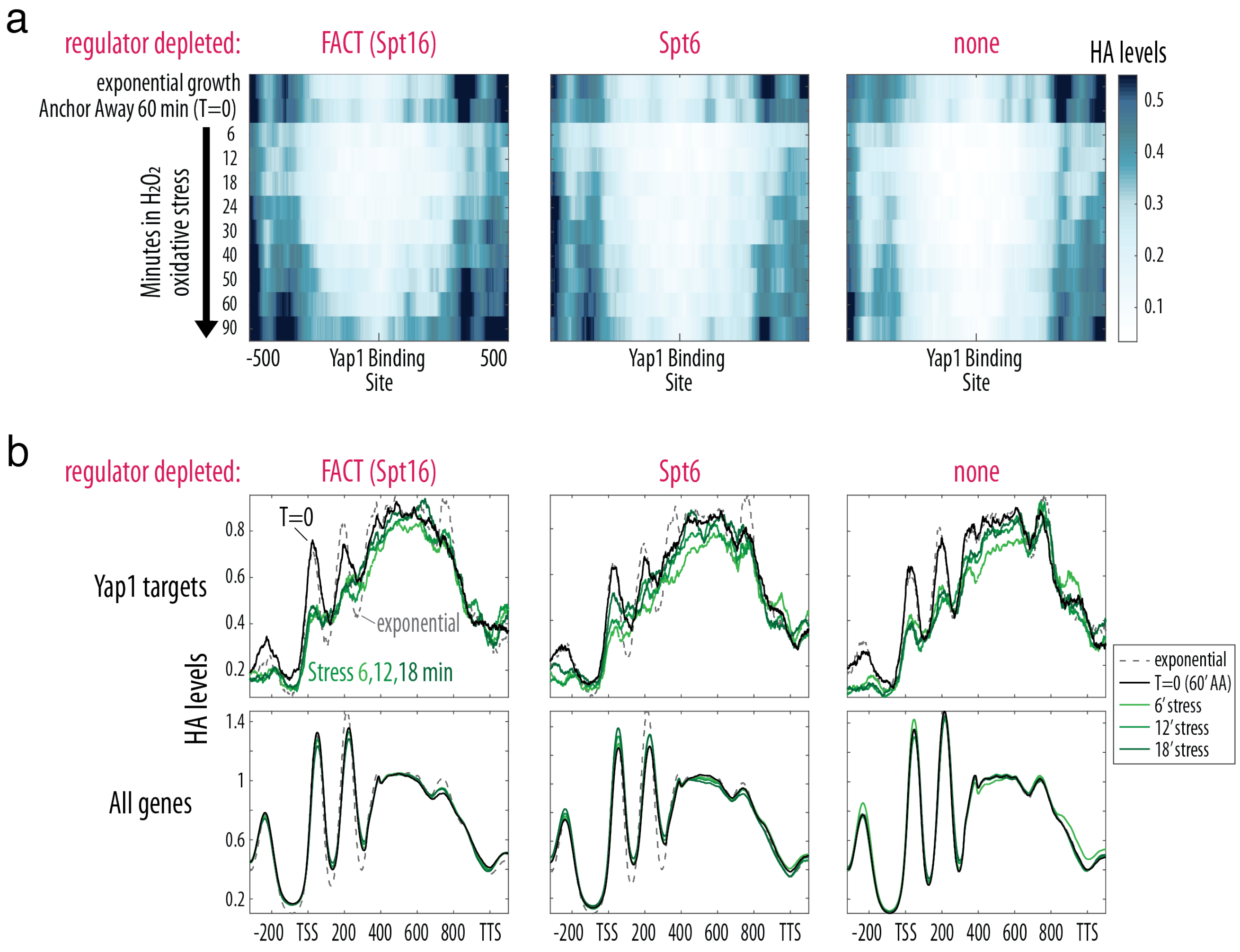

Figure N2. Occupancy changes in the promoter and beginning of the gene body following transcriptional reprogramming. Cultures were grown to exponential phase and supplemented with rapamycin for Anchor Away (AA) depletion of FACT (Spt16), Spt6 or no chromatin regulator. After 60 minutes of depletion (T=0), cells were stressed with 0.3mM H_2O_2 and followed for the indicated times by ChIP using HA to measure occupancy. **(a)** HA levels centered on binding sites of Yap1, a transcription factor specifically activated by oxidative stress (n=73), with 500 bp up and downstream shown. **(b)** HA meta gene profiles for Yap1 targets (top) or all genes (bottom) are shown scaled and aligned on the Transcription Start Site (TSS) of pre-stress and 6-18 minutes post-stress. Note the histone regulator-independent occupancy decrease at the promoter and first gene body nucleosomes following stress specifically at target genes.

4. As an additional test, we wished to internally assess our occupancy measurements within the samples analyzed and presented in the current manuscript. For this, we plotted HA levels as a function of gene expression level, where we have previously (Fig 3C in (2)) detected a signature of decreasing occupancy starting at the top 10% expressed genes. Here too, we detect the expected effect, localized to the highest expression levels (Fig N3, S4B).

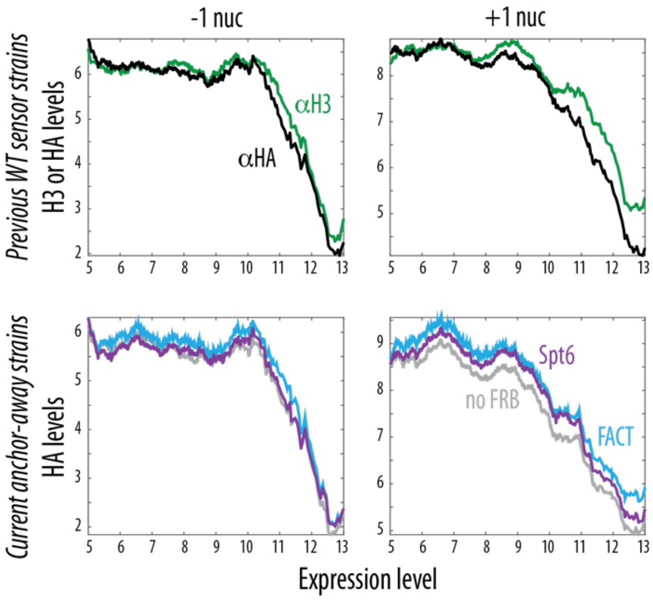

Figure N3. Occupancy changes as a function of expression levels. Occupancy on nucleosomes +1 or -1 was analyzed as a function of increasing expression levels in the original Hht2 allele sensor strain (top, assayed with HA or a total H3 antibody), and in strains in the current study derived from this strain with Spt16, Spt6 or no regulator (no FRB) Anchor Away modifications (bottom, data is shown for exponentially growing cells without regulator depletion). Note the highly similar signature across all strains and ChIPs of decreasing occupancy with increasing expression levels starting at high expression levels (~10).

5. Finally, we note that we do detect a significant decrease in occupancy for Spt6 and FACT when the compensating general chaperones are co-depleted with them (Fig S5C).

References

1. Jonas,F., Yaakov,G. and Barkai,N. (2022) Rtt109 promotes nucleosome replacement ahead of the replication fork. *Genome Res.*, **32**, 1089–1098.
2. Yaakov,G., Jonas,F. and Barkai,N. (2021) Measurement of histone replacement dynamics with genetically encoded exchange timers in yeast. *Nat Biotechnol*, **39**, 1434–1443.
